# SLUG: Vision-Based UAV Self-Localization in GNSS-Denied Urban Environments

**DOI:** 10.3390/s26196030

**Published:** 2026-09-23

**Authors:** Xuxu Qi, Enhui Zheng, Beicheng Li, Chen Tian, Xuehao Huang

**Affiliations:** School of Mechanical and Electrical Engineering, China Jiliang University, Hangzhou 310018, China; s24010811023@cjlu.edu.cn (X.Q.); p24010854052@cjlu.edu.cn (B.L.); s21010802008@cjlu.edu.cn (C.T.); s24010811012@cjlu.edu.cn (X.H.)

**Keywords:** UAV, cross-view visual geo-localization, transformer, image retrieval, CMHUAV dataset, HNDM@K

## Abstract

In complex environments such as cities and canyons, cross-view matching of UAV and satellite imagery serves as a complementary approach to self-localization in the absence of GNSS signals. To address the limitations of existing datasets—namely their limited variety of scenes and low annotation accuracy—we have constructed the Cross-View, Multi-Altitude, Multi-Scene UAV Localization Dataset (CMHUAV). This dataset covers nine adjacent, non-overlapping subregions in Hangzhou and comprises 8625 precisely matched pairs of drone and satellite images, providing high-precision supervision for cross-view feature learning. Furthermore, we designed a Transformer-based global feature enhancement localization model (SLUG) equipped with a lightweight Adaptive Part Weighting (APW) module, which learns part-level scalar importance weights to dynamically recalibrate the contribution of each annular region. By integrating adaptive part-level weighting with a joint supervision strategy, we significantly enhance SLUG’s spatial discrimination capability. We also propose the Height-aware Normalized Distance Matching (HNDM@K) metric, which unifies retrieval ranking quality and geospatial bias within a single framework, enabling joint evaluation of elevation-adaptive localization accuracy and retrieval performance. Experiments demonstrate that, on the CMHUAV dataset, SLUG achieves a recall of 93.53% and a mean precision of 93.9%; under the HNDM@1 metric, the localization scores at all three altitude levels exceed 94.8%. These results demonstrate that the proposed method, evaluated via offline visual retrieval, exhibits strong potential as a vision-based localization module for UAVs under GNSS disruption. Nevertheless, closed-loop flight tests and system-level localization performance still require further validation.

## 1. Introduction

Unmanned aerial vehicles (UAVs) are widely used for aerial photography in fields such as land surveying, urban management, emergency response, and autonomous navigation [1,2,3,4]; with improvements in onboard computing power and the emergence of lightweight vision models, vision-based UAV localization [5,6] has become a key area of research in the field of low-altitude autonomous navigation [7]. However, in complex environments such as urban canyons, GPS [8] signals are prone to obstruction or interference, leading to failures in autonomous drone localization. Cross-view visual geolocation [9] technology, which matches images captured by the drone downward-looking with satellite base maps, serves as an important supplementary method when GNSS [10,11] signals are obstructed.

As shown in Figure 1, the envisioned UAV self-localization framework consists of the onboard camera capturing real-time images, from which deep vision features are extracted using a Transformer network [12]; simultaneously, all reference images in the satellite baseline map have been pre-processed offline using the same Transformer model to form a feature database. During the online phase, the similarity between the UAV image features and those in the feature database is measured, and the top 10 satellite images with the highest similarity are selected as candidate matches. This process supports two positioning modes: In global positioning mode, the UAV images are compared one by one against the entire map feature library; this is suitable for coarse position estimation when the initial position is completely unknown. In local positioning mode, the actual or estimated GPS coordinates from the previous time step are utilized to select reference images for matching only within the geographical vicinity of those coordinates; this is suitable for efficient and precise positioning when the current approximate position is known. The two modes can be switched flexibly depending on the availability of actual GPS information, thereby balancing the robustness and real-time performance of positioning.

The DenseUAV [13] dataset, designed to meet the requirements for continuous autonomous localization by UAVs, has pioneered a new paradigm for constructing datasets through high-density sampling in real-world scenarios, providing a key reference for this study. However, this dataset has two limitations: firstly, the RTK (Real-Time Kinematic) module was not utilized during data collection, resulting in limited GPS annotation accuracy (at the meter level); secondly, it covers only a single campus setting and lacks diverse urban scenarios such as city roads, residential areas, and commercial districts, which can easily lead to model overfitting and insufficient robustness [14]. Existing cross-view localization datasets, such as University-1652 and SUES-200 [15], have limited scene coverage and low GPS annotation accuracy, which hinders model generalization and validation in real-world urban environments.

To address these issues—namely, the limited scene coverage of existing datasets, insufficient annotation accuracy, the challenges of feature alignment across views and heights, and the inability of traditional metrics to quantify spatial localization errors—this paper proposes a systematic solution encompassing dataset construction, model design, and evaluation metrics [16]. The main contributions are threefold:

(1) We construct CMHUAV, a cross-view, multi-altitude, multi-scenario UAV localization dataset. This work introduces a new benchmark that combines RTK-level annotations, three altitude levels, and nine diverse scenes, thereby providing a more challenging and comprehensive testbed for cross-view geo-localization.
(2) We propose a Transformer-based global feature enhancement localization model (Self-Localization with Unified Global features, SLUG) featuring a lightweight Adaptive Part Weighting (APW) head. Unlike the standard LPN that treats all annular regions equally, APW learns part-level scalar importance weights to dynamically calibrate part-level discriminability with negligible extra computational cost. Combined with the proposed adaptive part weighting and a joint CE+SWTri+KL supervision scheme, SLUG effectively enhances fine-grained spatial discrimination in high-density continuous sampling scenarios, addressing the core challenge of feature alignment across different viewpoints and altitudes.
(3) We propose HNDM@K, which overcomes the limitation of traditional Recall@K [17] and Average Precision (AP) [18] by integrating retrieval ranking quality with geospatial deviation into a unified framework. This enables joint quantification of localization accuracy and retrieval performance, providing a more engineering-oriented offline evaluation benchmark for UAV localization.

The remainder of this paper is structured as follows. Section 3 describes the CMHUAV dataset, the proposed SLUG model, and the evaluation metric HNDM@K. Section 4 presents the experimental setup and results, followed by an in-depth discussion. Section 5 concludes the paper and outlines future work.

## 2. Related Work

This section provides a systematic review of existing research in the field of cross-view visual geolocation, categorizing the relevant work into two main categories: cross-view geolocation [19] reference datasets and deep geolocation methods.

### 2.1. Geolocation Datasets

Cross-view matching methods can be broadly categorized into two main types. In the ground-satellite domain, early research pioneered the use of publicly available ground and satellite imagery to generate paired data consisting of ground-level and bird’s-eye-view images. Subsequently, the CVUSA [20] dataset expanded upon this by introducing ground-level panoramic images and satellite imagery, while CVACT [21] incorporated orientation a priori information. More recently, VIGOR [22] abandoned the assumption of center alignment, permitting queries at any location within a region.

In the drone-satellite domain, the University-1652 team was the first to introduce the drone’s perspective as an intermediate viewpoint between ground and satellite, reformulating the problem as an image retrieval task [23]. Subsequently, the SUES-200 dataset was collected at four different altitudes to address scale variations, while DenseUAV—designed specifically for continuous autonomous localization—adopted a novel high-density sampling paradigm for real-world scenarios.

Inspired by the research methodology of the DenseUAV dataset, this paper addresses the limitations of existing datasets—namely, their narrow range of scenarios and limited GPS annotation accuracy—by constructing the CMHUAV dataset. This dataset covers a wide variety of scenarios and flight altitudes, thereby further expanding the boundaries of the drone-satellite cross-view localization task. An overview of the existing geographical location data table is shown in Table 1.

### 2.2. Supervised Learning-Driven Approaches

The core idea behind this class of methods is to enhance the model’s ability to distinguish between features by designing various forms of supervisory signals [24] and loss functions. Cross-entropy loss [25], used as a supervisory signal, was the primary method employed in early convolutional neural networks (CNNs [12]). To improve the robustness of cross-view matching, triplet loss [26] has been widely adopted for this task; simultaneously, contrastive loss [27] has further enhanced matching accuracy by imposing more flexible constraints on positive and negative samples. Furthermore, methods related to multi-task supervised learning have also been introduced into this field.

### 2.3. Approach Guided by Matching Optimization

Cross-view localization methods aim to address three major challenges: spatial asymmetry, perspective distortion, and feature misalignment. Two distinct strategies have emerged to date. One strategy employs spatial transformations to reduce cross-domain feature differences; Dai, Shi et al. [28,29] applied polar coordinate transformations, while Toker et al. [30] utilized generative adversarial networks (GANs) to generate views of the target domain. The other strategy explicitly addresses the issue of feature misalignment: LPN [31] relies on block cropping, while FSRA [1] utilizes heatmap-based automatic region selection to achieve instance-aware segmentation. SAFA [32] further enhances region-level representation through spatial-aware feature aggregation. Although SAFA was not originally designed as a Transformer-based method, its spatial-aware aggregation mechanism is closely related to the region-wise matching problem considered in this work. In our experiments, SAFA is re-implemented under the same ViT-S/224 backbone as all other compared methods, so that the comparison focuses on the aggregation and matching strategy rather than the backbone architecture.

A unified feature representation is crucial for cross-view geolocation; in this process, the model adaptively aligns images from different visual domains to overcome distortions and scale changes caused by differing viewpoints. Autonomous visual localization tasks using UAV not only inherit the core challenges of traditional cross-view geolocation but also face greater geographical variations; they require simultaneous resolution of dynamic scene changes introduced by the temporal dimension and the precise capture of relative spatial positions. This constitutes the core optimization focus of this study.

Recent Transformer-based methods such as TransGeo [33], GeoFormer [34], and Sample4Geo [35] have further advanced cross-view feature alignment by leveraging pure self-attention, geometric priors, and hard sample mining, respectively. However, these methods primarily focus on global feature aggregation or fixed part partitioning, and they do not adaptively recalibrate part-level contributions under altitude-induced scale changes. For example, GeoFormer employs a Siamese Transformer for UAV geo-localization but still relies on global feature aggregation or fixed part partitioning. Our proposed APW module explicitly addresses this gap by learning part-level scalar weights to dynamically recalibrate each annular region under varying flight altitudes.

## 3. Preparations and Methodologies

### 3.1. CMHUAV Dataset

This section begins by outlining the main features of the cross-view, multi-altitude, multi-scenario UAV localization dataset (CMHUAV) developed in this paper, highlighting its key differences from existing publicly available datasets, before going on to describe in detail the dataset’s sampling methods and overall structure.

#### 3.1.1. Key Features

The core design objective of the CMHUAV dataset is to address the challenge of autonomous visual localization for drones when they encounter GPS signal interference in complex urban environments. All drone imagery was captured using a single downward-facing gimbal camera mounted on a DJI Mavic 3T, to maintain a bird’s-eye view consistent with the satellite base map. The platform is also equipped with a variety of onboard sensors, including a GNSS receiver, a RTK module, a barometer, an IMU, and visual obstacle avoidance cameras. Among these, the RTK module (in conjunction with GNSS) provides precise longitude, latitude, and altitude information for each image captured; these data are used solely for geotagging the drone images. Compared with existing datasets, this dataset was specifically designed with the following three aspects in mind.

From “single-point landmarks” to “continuous coverage across multiple scenarios”. Most existing datasets employ a discrete sampling approach based on isolated landmark buildings, resulting in significant visual differences between samples and making retrieval relatively straightforward. CMHUAV selected nine adjacent subregions that do not overlap spatially within Zhejiang Province, China (encompassing campuses, residential areas, commercial districts, and urban roads), within each region, adjacent sampling points have a fixed image overlap ratio. This design compels the model to learn fine-grained spatial relative positions within homogeneous scenes, thereby more closely approximating the real-world challenge of ‘precise positioning in similar environments’ encountered during continuous drone flight.

From “meter-level coarse labeling” to “RTK [36] centimeter-level ground truth”. Existing cross-view datasets generally suffer from substantial GPS annotation errors, leading to misaligned positive sample pairs and training noise that interferes with the model. CMHUAV employs the DJI Mavic 3T’s RTK high-precision positioning module for data acquisition, providing centimeter-level accurate geographic coordinates for each sampling point, thereby fundamentally eliminating label noise.

“Multi-altitude adaptation”. CMHUAV has specifically defined three relative ground heights—220 m, 200 m, and 180 m—to simulate positioning requirements at different resolutions and scales. The accompanying satellite base maps employ uniformly scaled high-resolution 2025 imagery, eliminating interference from temporal variations. The three altitudes yield different ground sampling distances (GSDs), approximately proportional to flight height, so that 180 m images contain more local texture while 220 m images provide wider contextual coverage. This deliberate design forces the model to learn scale-robust rather than resolution-specific representations.

To fully simulate the complexity and continuity of real urban environments, the collection areas cover diverse typical landforms including factories, residential districts, parks, schools, highway interchanges, and commercial areas (as shown in Figure 2). Multiple scenes are mixed within the same region, and urban roads are also included to prevent overfitting to a single campus scene. Figure 3 illustrates the flight path, flight direction and altitude settings for this dataset. The sampling points are marked with red circles, spaced 30 m apart, with the drone flying at altitudes of 180 m, 200 m, and 220 m above ground level, respectively. The camera direction is indicated by green arrows. The current, previous and next sampling points are denoted by T, T − 1, and T + 1, respectively. The overlapping areas are delineated by red dotted lines, while the illustration in the top right-hand corner shows the flight path from a bird’s-eye view, with black arrows indicating the direction of flight. The three altitude levels (220, 200, and 180 m) are marked by red, yellow, and green circles, respectively.

#### 3.1.2. Dataset Construction

For the CMHUAV dataset, drone image acquisition centered on three key aspects. At each sampling point, images were captured from both satellite and drone perspectives at three altitude levels (180 m, 200 m, and 220 m); Furthermore, we emphasized temporal diversity by sampling at different times between 06:00 and 18:00 under various lighting conditions, thereby increasing scene diversity. In addition, all data collection was carried out using a standardized downward-facing camera configuration. Flight path planning was performed using DJI’s flight control system, employing equidistant sampling and a fixed sampling interval.

For the accompanying satellite base maps, the CMHUAV dataset employs high-resolution satellite imagery from 2025, with all base maps precisely geo-aligned to the UAV sampling points. Unlike existing datasets that use multi-temporal satellite image slices, this dataset adopts satellite imagery from a single temporal dimension, focusing on the core challenge of cross-view and cross-altitude matching.

In terms of overall composition, all samples in CMHUAV are collected from nine adjacent, non-overlapping subregions within Zhejiang Province, China, covering typical urban scenes such as campuses, urban roads, residential areas, and commercial districts. For each of the nine acquisition regions, the sampling viewpoints were randomly divided into training and query subsets with a 70/30 ratio. The training subsets from all nine regions were then combined to form the overall training set, and the query subsets were combined to form the overall test set. Consequently, both the training and test sets contain viewpoints from every region, but no individual viewpoint appears in both splits. The training set comprises 6030 drone-view images and their corresponding 6030 satellite-view images, sourced from 2010 sampling points; the test set comprises 2595 images of each view type, sourced from 865 sampling points. Table 2 illustrates the composition of the data volume in the CMHUAV dataset.

All drone images were captured using a DJI Mavic 3T equipped with a network RTK module, and only images with RTK fixed-solution status were retained. The gimbal pitch angle was required to be within $3^{\circ }$ of nadir at the moment of exposure. The satellite base maps were 2025 high-resolution orthorectified imagery with a geometric correction error below 0.5 m (RMSE). UAV and satellite pairs were aligned in the UTM projection using the RTK-reported longitude and latitude at the time of capture. The main annotation error sources include RTK positioning error, satellite orthorectification error, and residual gimbal attitude error; after filtering, the combined horizontal annotation error is estimated to be less than 1 m (typically 0.3–0.6 m). Table 3 summarizes the key parameters.

### 3.2. The Benchmark Model Used

#### 3.2.1. Overall Framework

In the task of cross-view localization using drones and satellites, Dai et al. have demonstrated through systematic comparative experiments that models trained using a CNN-based backbone network perform significantly worse than those trained using a Transformer-based backbone network. To systematically investigate the effectiveness of different feature aggregation strategies [37], this paper constructs a unified baseline model framework. As shown in Figure 4, we adopt a dual-branch architecture based on shared weights as the main framework of our model, and we use ViT-S/224 as the unified feature extraction backbone network. The inputs consist of images from the UAV’s perspective and corresponding satellite views. All input samples undergo standardized data augmentation, followed by feature extraction via a shared backbone network. Subsequently, different head modules project the extracted features into a common retrieval space, while a multi-supervised loss function jointly optimizes the entire model.

#### 3.2.2. Data Augmentation Strategies

A consistent enhancement scheme is applied to both drone and satellite imagery, with the aim of improving the model’s robustness. Specifically, for drone direction enhancement, to diversify the flight directions of drones, we adopt a random rotation scheme where the maximum inscribed circle of the image is designated as the cropping boundary, and a square region is sampled from this at a randomly selected angle and then rotated [38]. For satellite viewpoint direction augmentation, random affine transformations [39] are applied to satellite images, introducing blank areas at the image edges caused by rotation, which enables the model to capture the global spatial context of satellite imagery. In addition, illumination robustness augmentation is performed via random brightness adjustments to accommodate brightness variations in UAV images captured under different times of day and weather conditions. Finally, to enhance robustness to spatial variations, random erasure [40] is introduced to simulate variations in the spatial distribution of objects caused by time shifts or dynamic scene changes.

#### 3.2.3. Shared Backbone Network

In the comparative experiments, this paper uniformly adopts ViT-S/224 as the shared backbone network for all comparison models. Benefiting from the self-attention mechanism, Transformer-based backbones exhibit superior capability in modeling long-range interrelations and global scene context. This advantage allows them to better handle the difficulties caused by cross-view domain gaps and altitude-induced spatial shifts. The use of a fixed, uniform backbone network minimises interference from differences in feature extraction capabilities, allowing subsequent performance differences to be attributed to variations in head design.

#### 3.2.4. Feature Aggregation Strategies

The prediction head is responsible for integrating the local tokens extracted by the backbone network into fixed-dimensional image-level retrieval features and constitutes a critical factor in determining the final localization accuracy. In order to determine the optimal feature aggregation strategy for autonomous positioning of drones across different viewpoints, we have systematically compared and evaluated three mainstream head designs within a unified framework.

Pooling-based heads. These heads generate global features by aggregating local tokens. For the feature maps output by ViT-S, category tokens are first extracted to serve as the basis for global features, after which the remaining local tokens are aggregated through various pooling operations. To further enhance the robustness of the features, we introduce Generalized Mean Pooling (GeM [41]) as the baseline head, which strikes a balance between feature extraction accuracy and computational efficiency through an adaptive global pooling strategy.

Feature-mapping aggregation heads. SingleBranch [13] and NetVLAD are both typical heads of this category.

SingleBranch adopts a single-branch linear transformation architecture, projecting and condensing all local tokens output by the Transformer into a unified dimension. With an extremely simple chain-like structure, it models the global representation while preserving original semantic information and controlling parameter count and computational cost.

NetVLAD [42], on the other hand, builds a visual dictionary with learnable clustering centers, performs soft assignment and residual aggregation for each local token, and exploits the semantic distribution relationships among tokens. Compared with plain pooling, it offers stronger fine-grained discrimination and is well suited for cross-view image retrieval tasks.

Chunking-based heads. To address the feature misalignment problem in cross-view images, this paper also evaluates head strategies based on region division. LPN reconstructs the ViT features into a 2D image format and, drawing on the idea of the Local Pattern Network (LPN) from CNN architectures, extracts features progressively from the image center to the edges in a patch-by-patch manner, enabling fine-grained spatial modeling. In FSRA, a heatmap-guided adaptive partitioning strategy is adopted, where the feature map is automatically segmented into multiple regions based on heatmap responses, rather than relying on manually predefined rules. This enables precise cross-view alignment and region-wise feature aggregation.

Based on the structural characteristics and design philosophies of the above three head types, and considering the core requirement of fine-grained spatial discriminability in the UAV cross-view localization task, this paper preliminarily selects chunking-based heads (LPN/FSRA-type) as the preferred direction. These heads, through explicit spatial partitioning or adaptive region alignment, can more effectively capture subtle visual differences between adjacent positions in continuous urban scenes, which aligns well with the design goal of the CMHUAV dataset—dense sampling that forces the model to learn fine spatial relationships.

To further enhance part-level discriminability, we propose a lightweight Adaptive Part Weighting (APW) module that learns part-level scalar importance weights and uses them to recalibrate the contribution of each annular region. The retrieval head equipped with this module is referred to as the APW head.

The complete localization model, termed SLUG, integrates the APW head with two additional components: (1) an asymmetric data augmentation strategy (Section 3.2.2) that adapts to the different characteristics of UAV-view and satellite-view images; (2) a joint loss function combining cross-entropy, soft-weighted triplet, and KL divergence (CE+SWTri+KL, Section 3.2.6).

Throughout this paper, APW denotes the retrieval head with the proposed weighting module, while SLUG represents the full model that combines APW with the asymmetric augmentation and the joint loss. Unless otherwise stated, all experiments involving APW use the same training configuration as other compared heads, and SLUG refers to the complete configuration.

By combining these architectural refinements with the overall optimization, the final ’Our Method’ baseline is anticipated to deliver notable gains in retrieval accuracy and cross-view matching robustness, while introducing only a slight rise in parameter count and computational expense.

To summarize, the above analysis identifies three categories of feature aggregation strategies. Among them, the chunking-based heads (LPN and FSRA) demonstrate superior fine-grained spatial discriminability, which aligns with the dense sampling nature of our CMHUAV dataset. However, these existing heads share a common limitation: they treat all partitioned regions equally during inference. Our proposed SLUG framework addresses this gap by introducing the APW module, which learns part-level scalar importance weights to dynamically recalibrate part-level features. This distinction—adaptive region-wise calibration versus fixed equal-weight aggregation—constitutes the primary methodological novelty of SLUG beyond simply combining existing components.

#### 3.2.5. Adaptive Part Weighting (APW) Module

Although the chunking-based heads (e.g., LPN and FSRA) effectively model fine-grained spatial structures via regional partitioning, they treat all partitioned regions with equal importance. In real-world UAV self-localization, however, different annular regions in the feature map contribute unequally to the final identity discrimination due to varying degrees of background clutter, occlusion, and altitude-induced scale changes. To address this limitation, we equip SLUG with a lightweight Adaptive Part Weighting (APW) module that learns part-level scalar importance weights and uses them to recalibrate the contribution of each part feature.

Specifically, given the two-dimensional feature map $X\in R^{B\times C\times H\times W}$ produced by the shared backbone, we first obtain a global context descriptor by applying global average pooling followed by a flattening operation, yielding $g=\mathrm{Flatten}\left(\mathrm{GAP}(X)\right)\in R^{B\times C}$. A weight generator G, consisting of two fully-connected layers with a ReLU activation and a final Sigmoid function, then produces the part-specific scalar weights, as shown in Equation (1):

(1) $$w=\sigma \left(\delta \left(gW_{1}\right)W_{2}\right),\;g\in R^{B\times C},\;W_{1}\in R^{C\times C},\;W_{2}\in R^{C\times K},\;w\in {\left[0,1\right]}^{B\times K}.$$

Here, $W_{1}\in R^{C\times C}$ and $W_{2}\in R^{C\times K}$ are learnable parameters (biases are omitted for simplicity), δ(·) and σ(·) denote ReLU and Sigmoid activations, respectively, and *K* is the number of annular parts (i.e., block). The resulting matrix w contains the part-specific scalar weights for each sample, where the *i*-th column corresponds to the weight of the *i*-th part.

In parallel, the LPN-style annular partitioning is applied to the same feature map X to produce *K* part descriptors. Each part descriptor is then passed through an independent ClassBlock, yielding a part-level classification logit $c_{i}$ and a part-level retrieval feature $F_{i}$. For a single sample, the learned scalar weight $w_{i}$ denotes the *i*-th element of w and is applied directly to the corresponding part feature, as shown in Equation (2):

(2) $$F_{i}^{\prime }=w_{i}\cdot F_{i},\;i=1,\ldots ,K.$$

Thus, the final part-level features $\left\{F_{i}^{\prime }\right\}$ are the weighted versions of the original part features. During training, all part-level classification logits $\left\{c_{i}\right\}$ are supervised by the cross-entropy loss, and the weighted features participate in the metric learning loss (e.g., soft-weighted triplet loss). At inference, the weighted part features are stacked with the global feature along a new dimension to form the final image representation.

The weight generator is trained jointly with the main cross-view retrieval loss, without requiring an auxiliary entropy loss or a separate training phase. This weighted aggregation emphasizes discriminative central and mid-annular regions while suppressing less informative outer regions that may contain background noise. Unlike attention mechanisms that compute weights from each part independently, APW derives all part weights from a single global context descriptor, allowing the network to compare the relative importance of different regions before assigning weights. The annular partition itself already provides multi-scale spatial context, from fine-grained central details to coarse outer layouts; APW learns to emphasize the most scale-relevant regions instead of treating all partitions uniformly. Compared with the standard LPN head, which simply concatenates equal-weight part features, the proposed APW module introduces lightweight instance-level feature recalibration, while avoiding the additional heatmap generation or complex region selection required by FSRA.

In our implementation, the number of annular regions (block) is set to 2 by default, which is selected based on the sensitivity analysis reported in Section 4.4.4. This choice provides the best trade-off between fine-grained spatial discrimination and computational cost. In the remainder of this paper, APW denotes the retrieval head equipped with the proposed weighting module, while SLUG represents the full localization model that integrates APW with the asymmetric augmentation strategy and the joint loss function.

#### 3.2.6. Design of a Unified Loss Function

To ensure fairness, all models adopt a unified multi-supervision loss system covering representation, metric, and mutual learning, which supervises classification, feature distance, and distribution alignment simultaneously.

Representation Learning: For classification supervision, the cross-entropy loss (CE) is employed [25]. A fully connected layer (FC) projects the extracted features into the class-label space, and the resulting logits are passed through Softmax to yield probability estimates. This arrangement drives the model towards learning class-discriminative representations.

Metric Learning: For optimizing feature-space similarity, the soft-weighted triplet loss (SWTri) serves as the core metric objective [26]. It encourages the embeddings of matching pairs to be close while ensuring that non-matching pairs remain far apart. Meanwhile, multi-scale adaptive weights $\lambda_{i}$ from LPN and FSRA are incorporated to dynamically adjust the gradient contribution of different sample pairs, handling hard samples more flexibly and significantly improving cross-view feature matching accuracy. The loss is defined as follows:

(3) $$L_{\mathrm{SWTri}}=\sum_{i=1}^{N}\lambda_{i}\cdot \max\left(d_{i,p}-d_{i,n}+\alpha ,0\right).$$

In this formulation, $d_{i,p}$ and $d_{i,n}$ denote the feature distances from the anchor to the positive and negative samples, respectively. The coefficient $\lambda_{i}$ is derived from the multi-scale adaptive mechanisms in LPN and FSRA. The hyperparameter α controls the margin in the WeightedSoftTripletLoss; a larger α imposes stronger penalties on hard negative samples, while a smaller α relaxes the triplet constraint. Its influence is further analyzed in the sensitivity study (Section 4.4.4). The alpha in the soft-weighted triplet loss is set to 3. This value was determined through a parameter sensitivity study (Section 4.4.4), which showed that alpha = 3 yields the best performance, while larger or smaller values degrade retrieval accuracy.

Mutual Learning: Bilateral distribution-level learning using the KL divergence loss (KLoss) is employed to reduce the distribution discrepancy between drone images and satellite images [43]. It computes the difference between the class probability distributions (after Softmax) of UAV and satellite branches. Through a bidirectional KL divergence constraint, the output distributions of the two branches are forced to become consistent, thereby improving the overall generalization performance. The loss is given by the following:

(4) $$L_{\mathrm{KL}}=\sum_{x}P\left(x\right)\log\frac{P(x)}{Q(x)}+\sum_{x}Q\left(x\right)\log\frac{Q(x)}{P(x)}.$$

where *P* and *Q* denote the class probability distributions from the UAV branch and the satellite branch after Softmax, respectively, and *x* indexes the classes. By simultaneously minimizing both the forward (*P* to *Q*) and reverse (*Q* to *P*) directions, this formulation enforces symmetric distribution alignment, effectively bridging the domain gap between cross-view images.

### 3.3. Evaluation Metrics

With regard to the cross-view self-localization task for unmanned aerial vehicles, this section begins by introducing metrics such as recall@K and AP, and it subsequently proposes the highly adaptive spatial distance metric HNDM@K, enabling a joint evaluation of retrieval performance and localization accuracy. Figure 5 presents a comparison between R@1 and HNDM@K (H = 200 m), covering the definitions of metrics, distance-weighted calculations, and the evaluation process. To provide a visual comparison of the differences between the various evaluation metrics, Figure 6 shows, on the left, comparison curves for recall, geographic distance, and HNDM@K, while the right-hand side provides typical matching examples for reference.

#### 3.3.1. Recall@K and AP Metrics

R@K measures whether the correct match appears among the top-K retrieved results. R@1 reflects single-shot localization accuracy, while larger K values indicate the model’s tolerance for candidate re-ranking in practice.

(5) $$\mathrm{Recall}@K=\frac{1}{\left|Q\right|}\sum_{q\in Q}I\left[g_{q}\in N_{K}\left(q\right)\right].$$

where $g_{q}$ denotes the ground-truth matched image for query *q*, $N_{K}\left(q\right)$ is the retrieved top-*K* candidate set for query *q*, and I[·] is the indicator function that returns 1 if $g_{q}$ belongs to $N_{K}\left(q\right)$ and 0 otherwise [44], with K∈{1,5,10} and Recall@1 being the strictest metric.

AP measures how well positive instances are ranked and is computed as the area under the precision–recall curve. Given a query *q*, its AP value is obtained as follows:

(6) $$AP\left(q\right)=\sum_{k=1}^{M}P\left(k\right)\cdot \Delta R\left(k\right),\;P\left(k\right)=\frac{1}{k}\sum_{i=1}^{k}y_{i},\;\Delta R\left(k\right)=R\left(k\right)-R\left(k-1\right)$$

where $y_{i}$ indicates whether the *i*-th retrieved result is a positive sample, R(k) denotes the recall for the top-*k* results, and *M* is the length of the retrieved list.

#### 3.3.2. HNDM@K

Recall-based metrics like Recall@K and AP@K are effective for evaluating retrieval ranking accuracy; however, they do not account for the spatial displacement between the estimated position and the actual location. Although the existing Spatial Distance Matching metric (SDM@K) [45] introduces geographic distance constraints, it adopts a fixed penalty parameter and thus cannot adapt to the varying spatial scales at different UAV flight altitudes, which affects the evaluation of localization errors. This metric is adopted as the main offline evaluation criterion for UAV self-localization in this work.

To address the limitations of these traditional metrics, HNDM@K is proposed, building upon the highly adaptive mechanism of SDM@K.

To account for the effect of different flight altitudes *h* on spatial scale, we define the altitude-adaptive attenuation coefficient α(h) as follows:

(7) $$\alpha \left(h\right)=s_{0}\cdot \frac{h_{\mathrm{ref}}}{h},$$

where the reference altitude is $h_{\mathrm{ref}}=200\;m$, and the baseline scaling factor is $s_{0}=5\times 10^{-3}$. This design ensures the following: when the flight altitude is low, the ground sampling distance (GSD) is small and spatial displacement is detected more sensitively, resulting in a higher penalty factor; when the flight altitude is high, the attenuation factor is reduced accordingly, thereby adaptively adjusting the evaluation criteria.

The distance $d_{i}$ is the ground separation between the query location and the *i*-th retrieved candidate, calculated using the Haversine formula:

$$d_{i}=2R\arcsin\left(\sqrt{\sin^{2}\left(\frac{\Delta \phi }{2}\right)+\cos\left(\phi_{q}\right)\cos\left(\phi_{i}\right)\sin^{2}\left(\frac{\Delta \lambda }{2}\right)}\right),$$

where *R* is the Earth’s radius, $\phi_{q},\lambda_{q}$ and $\phi_{i},\lambda_{i}$ are the latitudes and longitudes of the query and candidate locations, and $\Delta \phi =\phi_{i}-\phi_{q}$, $\Delta \lambda =\lambda_{i}-\lambda_{q}$ are their differences.

For the candidate at rank *i* (1≤i≤K) in the Top-*K* results, its score weight $w_{i}$ is defined as follows:

(8) $$w_{i}=\frac{K-i+1}{e^{\alpha \left(h\right)\cdot d_{i}}},$$

This weight consists of two factors: the ranking factor (K−i+1) assigns greater weight to higher-ranked results, while the exponential decay factor $e^{\alpha \left(h\right)\cdot d_{i}}$ smoothly penalizes candidates based on their geographic distance—the farther from the true location, the lower the weight. Together, they simultaneously consider both retrieval ranking quality and spatial localization accuracy.

Furthermore, to exclude excessively large distances and focus on the effective localization error range, we introduce a distance threshold *T* (set to T=50m in this paper). In computing HNDM@K, only candidates with $d_{i}\leq T$ contribute to the numerator. On this basis, the ratio of the sum of the weighted scores of the valid candidates to the theoretically maximum sum of weighted scores is considered to be HNDM@K:

(9) $$\mathrm{HNDM}@K=\frac{\sum_{i=1}^{K}x_{\left(d_{i}\leq T\right)}\cdot w_{i}}{\sum_{i=1}^{K}\left(K-i+1\right)}.$$

Here, $x_{\left(d_{i}\leq T\right)}$ returns 1 when the specified condition is satisfied and 0 otherwise. The denominator $\sum_{i=1}^{K}\left(K-i+1\right)$ represents the maximum possible sum of weighted scores under the ideal case (i.e., all $d_{i}=0$ and all candidates are valid). Thus, the range of HNDM@K is strictly confined to [0,1], with larger values indicating better overall localization performance. Here, the distance threshold *T* is fixed to T=50m. HNDM@K is therefore used directly as the primary evaluation metric throughout this paper. In this work, $d_{i}$ is the great-circle ground distance computed by the Haversine formula from the UAV-view query location to the *i*-th candidate location.

In particular, when T→∞, all candidates are included, and HNDM@K reduces to a version without threshold truncation, yet the normalization denominator remains the theoretical maximum, ensuring that the metric always lies in [0,1]. In this paper, we compute HNDM@K separately for each flight altitude, to comprehensively evaluate the model’s cross-scale localization error performance at different altitudes. A comparison of HNDM@1 and Recall@1 is shown in Table 4.

## 4. Experimental Results and Analysis

### 4.1. Experimental Setup

Experimental configurations for the model architecture were uniformly established based on the Timm ecosystem to ensure that consistent features were extracted from the backbone network. For optimization, the SGD [46] algorithm was employed—a standard choice in computer vision—with an initial learning rate of 0.003 and batch size of 16. A layer-wise strategy was introduced to prevent the pretrained backbone from being disrupted by large updates: the backbone received 0.3× the base learning rate, whereas the head was trained at the full rate. The whole procedure was run for 120 epochs, during which two decays (by a factor of 0.1) occurred at the 70th and 110th epochs. All images from both views were resized to 224 × 224 and normalized before feeding into the network. At the feature level, the deep features are projected into 512-dimensional embeddings, and finally the classification layer produces the prediction output.

To ensure a fair comparison, all compared methods—including the newly added Transformer-based methods—are trained with exactly the same hyperparameters as the SingleBranch baseline (e.g., learning rate 0.003, SGD optimizer, batch size 16, 120 epochs, block = 1 where applicable, and identical data augmentation). No method-specific tuning is performed. This isolates the effect of the retrieval head design from other factors. All methods are trained with the same joint loss (CE+SWTri+KL) as the SingleBranch baseline. The default hyperparameters for the proposed SLUG model are block = 2 and SWTri alpha = 3. These values are justified by the sensitivity analysis in Section 4.4.4.

All experiments in this section are offline image-retrieval evaluations using pre-collected UAV and satellite images. No closed-loop flight, real-time onboard inference, continuous trajectory localization, or accumulated drift evaluation are included.

### 4.2. Dataset Validation

A cross-dataset comparative experiment was carried out to verify the effectiveness of the CMHUAV dataset. Firstly, using the baseline model as a unified framework, we replaced its detection head with different variants and trained and tested these models on both the multi-scene, multi-altitude CMHUAV dataset and the single-scene DenseUAV dataset. Among all candidates, the model exhibiting the highest performance was chosen, which then underwent further fine-tuning and optimization to produce the final optimal model (SLUG).

Although the same model achieves higher absolute scores on CMHUAV than on DenseUAV, this cross-dataset comparison alone cannot isolate the contribution of annotation accuracy. The two datasets differ simultaneously in scene composition, sample distribution, annotation precision, altitude configuration, and data volume. Therefore, we do not interpret the observed improvement as evidence that RTK-level annotation is the sole or primary cause. Instead, CMHUAV serves as a complementary benchmark that provides centimeter-level ground truth, multi-scene coverage, and multi-altitude settings. The relative advantage of SLUG over the compared baselines is consistently observed within each dataset, which remains the main evidence for the effectiveness of the proposed method.

### 4.3. Comparison with Existing Methods

To systematically evaluate the impact of different feature aggregation strategies on model performance, all methods utilized ViT-S/224 [47] as the backbone network, and comparative experiments were conducted on the CMHUAV and DenseUAV datasets. Table 5 and Table 6 present the performance comparison of various methods on the CMHUAV multi-height multi-scene dataset and the single-scene DenseUAV dataset, respectively. The results demonstrate that SLUG achieves the best performance on both datasets, with R@1 scores of 93.53% and 88.03%, significantly outperforming the other compared methods.

The APW (block = 1) in this table follows the baseline configuration for fair comparison with existing methods; its performance under the full SLUG configuration is reported separately in the sensitivity analysis (Section 4.4.4).

To further compare with recent cross-view localization methods under a controlled protocol, we re-implement SAFA, TransGeo, GeoFormer, and Sample4Geo using the same ViT-S/224 backbone, the same training schedule, the same joint loss (CE+SWTri+KL), and the same baseline augmentation as the SingleBranch baseline. These methods are not trained with their official configurations or method-specific tuning. Therefore, the comparison in Table 7 isolates the effect of the retrieval/aggregation design under identical conditions and should not be interpreted as a state-of-the-art benchmark or as evidence that the proposed head outperforms these methods under their official settings. As shown in Table 7, within this unified re-implementation setting, APW (block = 1) achieves 92.33% R@1, which is 2.81 percentage points higher than the best R@1 among the re-implemented competing methods (GeoFormer, 89.52%). This result supports the effectiveness of adaptive part weighting for retrieval-head design under controlled conditions. Sample4Geo was originally designed with a complex multi-scale hard-sample mining strategy and a different training schedule; under our unified baseline configuration, it may not reach its optimal performance, and its result is included only for completeness under the same protocol.

### 4.4. Component-Wise Ablation Study

To comprehensively evaluate the independent contribution of each proposed component, we conduct systematic component-wise ablation studies under strictly controlled conditions. All experiments employ ViT-S/224 as the unified backbone.

#### 4.4.1. Effect of the APW Module

We first isolate the contribution of the proposed APW module by comparing the standard LPN (block = 2) head with our APW (block = 2) head under identical conditions, i.e., the same asymmetric augmentation strategy and the same joint loss (CE+SWTri+KL), with the only variable being the retrieval head design. As shown in Table 5 and Table 6, APW improves R@1 by 1.11 percentage points over LPN (from 91.41% to 92.52%) on the CMHUAV dataset and by 2.06 percentage points (from 84.51% to 86.57%) on the DenseUAV dataset in the Drone → Satellite direction. This cleanly quantifies the effectiveness of the proposed adaptive part-wise weighting mechanism in enhancing fine-grained spatial discriminability.

#### 4.4.2. Effect of the Asymmetric Augmentation Strategy

To evaluate the contribution of the asymmetric augmentation strategy, we compare three settings under the same backbone, APW head, and joint loss CE+SWTri+KL: no augmentation, symmetric augmentation, and our asymmetric augmentation. As shown in Table 8, removing augmentation reduces R@1 to 91.02%, while applying symmetric augmentation only slightly improves performance to 91.25%. In contrast, the asymmetric augmentation strategy raises R@1 to 93.53% and achieves the best HNDM@1 values across all altitudes. This confirms that adapting augmentation to the different characteristics of UAV-view and satellite-view images effectively bridges the cross-view domain gap.

#### 4.4.3. Effect of the Joint Loss Function

To evaluate the contribution of each loss component, we compare seven configurations under the same backbone, APW head, and asymmetric augmentation strategy: CE only, SWTri only, KL only, SWTri+KL, CE+KL, CE+SWTri, and the complete joint loss CE+SWTri+KL. As shown in Table 9, using KL alone yields near-random retrieval performance (R@1 = 2.7%), while using SWTri alone performs clearly worse than the CE-only baseline (R@1 = 81.73% vs. 89.63%). This indicates that the cross-entropy classification objective is essential for learning discriminative representations. Starting from the CE-only baseline, adding SWTri improves R@1 by 1.51 percentage points (89.63% to 91.14%), whereas adding KL improves it by 1.93 percentage points (89.63% to 91.56%). When KL is further combined with SWTri, it yields an additional gain of 2.39 percentage points over CE+SWTri (91.14% to 93.53%), and the complete joint loss achieves the best HNDM@1 values across all three altitudes. These consistent improvements demonstrate that KL acts as an effective distribution-alignment regularizer when combined with the primary discriminative and metric-learning objectives. Nevertheless, because KL alone performs near chance level, its role should be interpreted as auxiliary rather than as an independent supervision signal. Overall, CE provides the primary discriminative supervision, SWTri further improves metric learning, and KL serves as a supplementary regularizer that consistently enhances cross-view feature alignment when combined with the main objectives.

#### 4.4.4. Parameter Sensitivity Analysis

To verify that the reported improvements do not rely on a particular hyperparameter configuration, we conduct sensitivity experiments on two key parameters: (i) the number of annular regions (block) in the chunking-based head, and (ii) the alpha value in the WeightedSoftTripletLoss. All experiments in this subsection use the same backbone (ViT-S/224), asymmetric augmentation, and the complete joint loss (CE+SWTri+KL), with only the parameter under study being varied.

Table 10 summarizes the results. For the number of regions, the default configuration (block = 2) achieves the best overall performance on R@1 (93.53%) and AP (93.90%). Increasing the block to 3 yields nearly identical accuracy (93.49% R@1) with slightly improved HNDM@1 at 180 m and 220 m, indicating that a finer partition does not further benefit the dense urban scenario, while block = 1 leads to a clear performance drop (92.76% R@1). This confirms that a moderate spatial granularity (block = 2) balances local detail and global context, and the model is not sensitive to a small perturbation of this parameter.

For the alpha of WeightedSoftTripletLoss, the best performance is observed at alpha = 3 (93.53% R@1, 93.90% AP). Increasing the alpha to 5 or decreasing it to 1 both degrade performance, with alpha = 1 showing the lowest R@1 (91.45%). This demonstrates that the chosen alpha value properly adjusts the contribution of hard samples in the triplet constraint; a smaller alpha makes the constraint too lax, while a larger alpha over-penalizes hard positives and disturbs convergence.

Overall, these sensitivity results indicate that the default hyperparameters (block = 2, alpha = 3) are near-optimal, and the reported gains are robust within a reasonable range of these values.

#### 4.4.5. Summary

The systematic component-wise ablation studies presented above collectively demonstrate the independent and complementary contributions of the three key components in SLUG: (1) the asymmetric augmentation strategy contributes a +2.51% gain in R@1 by bridging the cross-view domain gap; (2) the APW module contributes a +1.11% gain by adaptively calibrating part-level discriminability; and (3) the joint loss (CE+SWTri+KL) achieves the highest R@1 and HNDM@1 among the tested variants, with a small additional gain over CE+SWTri. This suggests that KL provides a limited but beneficial regularization effect for cross-view distribution alignment, while CE and SWTri serve as the main supervision signals. The synergy among the three components is therefore better described as supplementary rather than strongly complementary.

### 4.5. Model Head Ablation

This section presents ablation results for different prediction head configurations. The ViT-S model serves as the unified backbone, with a learning rate of 0.003 and a chunk parameter of 2 across all runs. Quantitative outcomes are listed in Table 11, and the per-thousand-image inference cost is also provided. SLUG achieves a notable improvement in retrieval accuracy, with only a modest increase in parameter count and relative forward time (1.10× over the SingleBranch baseline). The APW module introduces approximately 1.2 M additional parameters, including the weight generator and the part-level classifiers.

Relative forward time is measured on the same server with the same software environment, batch size, input resolution, and numerical precision for all methods. It includes only the network forward pass of the backbone and retrieval head, and it excludes image decoding, data augmentation, satellite feature database construction, feature normalization, similarity search, top-K ranking, and I/O. Since the database similarity search is identical for all compared retrieval heads, it is not included in the head comparison. These values are not absolute latency and are not used to claim onboard real-time performance.

As shown in Figure 7, although the results vary across the three test heights and head configurations, the HNDM curves exhibit similar decaying trends with increasing *K*. Different heads maintain consistent relative performance ranking across 180 m, 200 m, and 220 m altitudes.

### 4.6. Geographical Generalization: Leave-One-Region-Out Evaluation

The CMHUAV dataset consists of nine acquisition regions. To evaluate the model’s performance on regions not used in training, we employed the “leave-one-out” method: in each iteration, one region was reserved as the test set, while the remaining eight regions were used for training. This process was repeated nine times, ensuring that each region was tested once. Table 12 and Table 13 present the results for SLUG and the baseline model (SingleBranch), respectively.

Since the data sizes across the nine experiments were similar, the average results from all nine experiments are presented below. On average, SLUG achieved an R@1 of 80.87% and an AP of 82.21%, representing improvements of 1.57 and 2.37 percentage points, respectively, over the baseline. At the three heights of 180 m, 200 m, and 220 m, SLUG’s average HNDM@1 values were 87.80%, 88.45%, and 88.45%, respectively, all of which were higher than the baseline.

Results varied significantly across different retention regions. Rot6 and Rot7 are relatively difficult regions, where SLUG’s R@1 was 74.19% and 72.98%, respectively—6.06 and 3.93 percentage points higher than the baseline; however, in Rot8, SLUG’s R@1 was 87.36%, slightly lower than the baseline of 88.34%. This indicates that SLUG demonstrates a clear advantage in most challenging regions, but it still exhibits some variability in individual regions.

Compared to the results of the original random split (SLUG R@1 = 93.53%, baseline R@1 = 88.09%), the average accuracy under the “leave-one-out” split shows a significant decline. Because the random split is performed within each region, training and query viewpoints from the same region may be spatially adjacent. This can lead to over-optimistic evaluation results compared with the leave-one-region-out protocol, where the test region is completely unseen during training.

The training and test sets of the DenseUAV dataset come from different school campuses that do not overlap spatially, serving as a reference under conditions of no spatial overlap. As shown in Table 6, SLUG achieves an R@1 of 88.03%, a 5.02 percentage point improvement over the baseline (83.01%), indicating that SLUG maintains its advantage even when the training and test regions do not overlap, and that the performance improvement does not rely entirely on memorization of the training region. However, the gap between the CMHUAV random partitioning results and the leave-one-region-out results suggests that random partitioning may have contributed to part of the gain, though the exact proportion is difficult to quantify directly.

Overall, the leave-one-region-out experiments and the DenseUAV cross-campus evaluation indicate that SLUG possesses a certain degree of geographical generalization advantage over the baseline, but its advantage in completely unseen urban areas still needs further validation. Future work will include cross-city and cross-dataset transfer experiments to further evaluate its generalization capabilities.

### 4.7. Interpretation of Experimental Results

A comparison between CMHUAV and DenseUAV shows a higher R@1 on CMHUAV when using the same model (93.53% vs. 88.03%). While this may be partially attributed to the cleaner supervision provided by RTK annotations, it is important to note that the two datasets also differ in scene composition, sample distribution, altitude configuration, and data volume. Therefore, this cross-dataset comparison should not be interpreted as causal evidence that annotation accuracy alone determines performance. Instead, it suggests that high-quality annotations and diverse scenes are both important factors in cross-view localization, but their individual contributions cannot be separated without controlled experiments. The consistent improvement of SLUG over the baseline within each dataset remains the more reliable indicator of the proposed method’s effectiveness.

Necessity of fine-grained spatial feature modeling. The region-based LPN and FSRA methods achieved an improvement of nearly 3.7 percentage points on the R@1 metric compared with the globally aggregated methods. This gain fully validates the critical value of fine-grained spatial feature modeling in UAV cross-view localization tasks. In the continuously sampled scenes of CMHUAV, where adjacent sampling points exhibit extremely subtle visual differences, global features are insufficient for effective discrimination, whereas fine-grained features provide crucial discriminative cues.

Synergistic effects of loss function components. Ablation experiments demonstrate that using only the CE loss yields limited discriminative capability; the introduction of SWTri metric loss significantly boosts performance, proving that the soft-weighted triplet constraint enhances the discriminability of the cross-view feature space. To align the feature distributions of the UAV and Satellite branches, we incorporate KL divergence as a regularization component that encourages mutual learning between the two views. These results indicate that CE and SWTri are the primary supervision signals, while KL acts as a supplementary regularizer with a limited incremental effect.

The effect of flight altitude on localization performance can be explained from three aspects. First, according to the GSD relation, lower-altitude images have smaller ground sampling distances and therefore contain finer local textures but weaker global context; higher-altitude images exhibit the opposite. This scale difference directly affects the discriminative cues available for matching. Second, the per-altitude HNDM@1 results in Table 5 show that SLUG consistently achieves higher accuracy at 220 m than at 180 m, indicating that larger spatial context helps reduce visual ambiguity in dense urban scenes. Third, the proposed APW module adaptively recalibrates annular part weights according to the input; although the learned weights are not explicitly conditioned on altitude, the training distribution enables the weighting mechanism to favour fine-grained central regions when local details are informative (low altitude) and broader annular regions when global layout is more reliable (high altitude). This altitude-adaptive behavior, even without explicit altitude input, explains why SLUG maintains robust performance across the entire 180–220 m flight envelope.

### 4.8. Offline Diagnostic Analysis of Evaluation Metrics

Clarification on the role of HNDM@K. HNDM@K requires the ground-truth geographic coordinates of the UAV query to compute the Haversine distance $d_{i}$ to each retrieved candidate. It is therefore an offline evaluation metric. The HNDM-guided selection and the R@1+HNDM@1 fusion reported below are offline oracle analyses used to diagnose metric behavior; they are not online candidate-selection or localization strategies under GNSS-denied conditions. They become usable online only if an independent source of query-position information is available, such as a previous filtered estimate, visual-inertial odometry, or another auxiliary sensor. Otherwise, HNDM@K should be regarded solely as an offline evaluation metric.

To illustrate how incorporating geospatial distance affects metric behavior in an offline diagnostic setting, we compare three protocols on the CMHUAV test set under different distance thresholds: (1) Pure visual R@1: The predicted location is simply the geotag of the top-1 retrieved satellite image; this is the only online-deployable protocol among the three. (2) HNDM-guided selection (offline oracle): Among the top-*K* retrieved candidates, the one with the highest HNDM score is selected using the ground-truth query coordinates. (3) Fusion strategy (offline oracle): The final location is determined by fusing visual similarity and the HNDM score of the top-*K* candidates; this also requires the ground-truth query coordinates.

Table 14 and Figure 8 show the spatial distributions of predicted and true positions for these three protocols. In this subsection, HNDM@1 denotes the HNDM metric computed with the distance threshold *T* set to the indicated value. The terms “HNDM-guided” and “Fusion” refer only to offline diagnostic protocols, not deployable online strategies.

High-precision localization scenarios. At a distance threshold of 1m, pure visual R@1 achieves an accuracy of 0%, while the offline HNDM-guided protocol attains an accuracy of 1.96%. As the threshold is relaxed to 3m and 5m, HNDM@1 rapidly climbs to 11.76% and 34.31%, respectively, which are four times and three times the corresponding R@1 values. This gap reveals the core pain point of pure visual methods in close-range localization: the model may have “recognized the right scene” but fails to “locate the exact position”.

Medium-distance localization scenarios. When the threshold is raised to 10m, HNDM@1 achieves an accuracy of 84.31%, already approaching the level of pure visual R@1 at the 100m threshold (88.24%). As the threshold is further relaxed to 20m, HNDM@1 reaches 99.02%; at thresholds of 30m and above, HNDM@1 fully attains a matching accuracy of 100%.

Performance bottleneck of pure visual methods. Pure visual R@1 achieves only 88.24% accuracy at the 100m threshold, with nearly 12% of query samples failing to match any spatially proximate satellite image. This 12% “localization blind zone” is a systemic issue caused by visual ambiguity—factors such as symmetric structures, repetitive textures, and homogeneous scenes prevent the model from distinguishing the true location from distant “visual clones” in the visual feature space.

Core advantage of HNDM@K as an offline evaluation metric. HNDM@K changes the offline evaluation paradigm—from “does this image look alike?” to “is this position close enough?”. It is not an online candidate-selection rule, because computing the distance penalty requires the ground-truth query coordinates. In complex scenes where visual features are highly similar, HNDM@1 calculates the great circle distance using the Haversine formula and applies an exponential penalty that adjusts with altitude, thereby assigning zero or low weight to visually similar but geographically distant candidates during offline evaluation. The longitude and latitude coordinates required for this computation can be directly obtained from the metadata of the satellite base maps, without the need for additional sensors or prior map information.

### 4.9. Visualization and Failure Case Studies

In the sample-level visualization results of the baseline model for the localization task, the drone images also show differences in brightness and capture altitude across different time points. As can be clearly seen from Figure 9, drone images and satellite images differ in terms of both domain features and spatial structure. At the same time, as the acquired images cover contiguous urban areas, highly similar samples frequently occur, which may lead to localization results pointing to similar yet entirely distinct regions. Although we employed metric learning and mutual learning strategies to mitigate the domain gap, some queries still fail to be correctly matched with the corresponding satellite images.

The traditional Recall@1 metric only measures whether a positive sample is hit in the retrieval results, without reflecting the spatial deviation of localization. Figure 10 presents four typical failure cases, where Recall@1 exhibits obvious limitations. In contrast, our proposed HNDM@1 metric integrates retrieval ranking with geographic distance constraints and incorporates an altitude-adaptive mechanism, effectively alleviating these limitations.

Figure 10 illustrates four key challenges and their corresponding solutions based on HNDM@K. Confusion between spatially adjacent positions: As shown in Figure 10a, dense sampling leads to high visual similarity between UAV images of adjacent locations. Recall@1 only cares about whether the retrieval result “hits” a satellite image with the exact same geographic coordinate and is unable to distinguish misclassifications to neighboring positions. In contrast, HNDM@1 applies a geographic distance-based exponential penalty, assigning lower scores to retrieved results that are spatially close but not the correct location. Viewpoint shifts and spatial inconsistency at the same geographic coordinate: As shown in Figure 10b, variations in viewpoint, illumination, and time cause inconsistent image features for the same coordinate. Recall@1 lacks tolerance for such cross-view discrepancies. HNDM@1 incorporates a continuous geographic distance decay, allowing a certain range of feature drift. As long as the retrieved result falls within an acceptable error threshold, it still receives a high score. Localization ambiguity in symmetric scenes: As shown in Figure 10c, symmetric structures are prone to mirror mismatches. Recall@1 treats the mirrored location as a “false” result, but fails to quantify the geometric deviation from the true location. HNDM@1 evaluates the spatial deviation between the search results and the true location using the great circle distance, while significantly reducing the weighting of prominent deviations caused by mirror symmetry. Cross-location mismatches in homogeneous similar scenes: As shown in Figure 10d, similar scenes at different geographic locations are visually indistinguishable. Recall@1 only outputs a binary “right/wrong” decision without reflecting the degree of mismatch. By contrast, the hierarchical scores generated by HNDM@1 are determined by the actual spatial offset between the predicted and true locations.

In summary, the traditional Recall@1 metric struggles to accurately measure the model’s actual localization capability in the four types of scenarios described above. As an offline evaluation metric, HNDM@K incorporates geospatial distance and an altitude-adaptive penalty, enabling a joint evaluation of retrieval accuracy and localization error. It provides a fairer and more fine-grained reflection of the model’s comprehensive localization ability, without implying that HNDM-guided candidate selection is directly deployable online.

### 4.10. Discussion on Practical Deployment and Limitations

#### 4.10.1. Scope, Deployment Boundary, and Key Limitations

Scope clarification. This work is an offline, single-frame image-retrieval study. It does not perform onboard deployment, closed-loop flight, continuous trajectory estimation, or drift evaluation. All experiments are offline evaluations using pre-collected UAV images and a pre-built satellite database. The relative forward time in Table 11 compares retrieval heads under identical server-side conditions and excludes database search, top-K ranking, and I/O; it must not be interpreted as onboard or end-to-end latency. We do not claim that the proposed method solves deployment-level challenges; they are explicitly listed as limitations and future work.

Data hunger and structural changes. The method relies on a large number of UAV–satellite pairs for training. CMHUAV provides 6030 training pairs with RTK-level annotations, and standard augmentation plus an ImageNet-pretrained ViT-S backbone is used to reduce data demand. However, a systematic low-data ablation is not included. In addition, the current method assumes a static satellite base map; if large buildings are demolished, construction sites appear, or the urban layout changes significantly, the global context may be disturbed. CMHUAV uses a static 2025 base map, so this issue is not evaluated. Future work includes low-data training, base-map updating, change detection, and temporal fusion.

Real-time latency and drift. For future UAV deployment, weight, size, and power are strictly limited. The ViT-S/224 backbone contains about 22 M parameters and 8.5 GFLOPs; it could potentially be compressed via distillation, pruning, and INT8 quantization for embedded platforms such as NVIDIA Jetson, but this has not been validated here. Satellite features can be pre-extracted offline, and the two-stage localization strategy can restrict the search range to the previous estimate in local mode. These are conceptual considerations only; onboard latency and power benchmarking remain future work. We also do not claim drift elimination: single-frame retrieval cannot estimate or correct accumulated drift, which requires temporal filtering, VIO/IMU fusion, and closed-loop flight tests.

HNDM@K as an offline metric. HNDM@K is an offline evaluation metric rather than an online candidate-selection rule, because computing $d_{i}$ requires the ground-truth query coordinates. In future onboard systems, if an independent position estimate is available from VIO/IMU/filtering, HNDM-like scores may be used for candidate re-ranking; otherwise, HNDM@K should remain an offline benchmark.

Coverage of the present ablation study. The ablations in Table 5, Table 6, Table 7, Table 8, Table 9, Table 10 and Table 11 cover all components proposed and evaluated in this offline study, including the APW module, asymmetric augmentation, joint loss, retrieval heads, and key hyperparameters. Deployment-level ablations for data hunger, structural changes, and onboard latency would require additional datasets, onboard hardware, and closed-loop flight tests, and are therefore left as future work.

#### 4.10.2. Generalization and Scope of Comparison

Generalization across scenes, seasons, and urban changes. Although CMHUAV covers nine diverse urban scenes, it was collected under favorable lighting and clear weather. Seasonal variations, adverse weather, low-light conditions, and long-term infrastructure changes are not represented. Under such domain shifts, performance is expected to degrade. Future work will investigate style-transfer-based generative augmentation and regular base-map updates with IMU/barometer dead-reckoning fallback.

Scope of the unified re-implementation comparison. The comparison in Table 7 is a controlled study of feature aggregation and retrieval heads, not an official benchmark. Because TransGeo, GeoFormer, Sample4Geo, and SAFA were re-implemented without their method-specific backbones, input resolutions, pretraining strategies, losses, augmentation policies, or training schedules, the reported numbers may differ from their original papers. All conclusions from Table 7 are limited to the present unified configuration. We do not claim that the proposed APW head is superior to these methods under their official configurations.

## 5. Conclusions

Our systematic study of UAV visual autonomous localization under GNSS-denied conditions is organized around three pillars: the dataset, the network architecture, and the evaluation methodology. In terms of the dataset, we construct the CMHUAV cross-view, multi-altitude, multi-scenario localization dataset, which organically integrates RTK centimeter-level ground-truth measurements, adaptability across three altitude levels, and nine adjacent, non-overlapping subregions covering diverse continuous urban scenarios, thereby addressing the dual deficiencies of existing datasets in annotation accuracy and scenario diversity. In terms of the model, we propose a global feature-enhanced Transformer localization model (SLUG) equipped with a novel APW module. The APW module learns part-level scalar importance weights to dynamically recalibrate the contribution of each annular region, effectively suppressing background clutter and emphasizing discriminative spatial cues with negligible extra computational cost. By integrating APW with multi-scale adaptive aggregation and a joint CE+SWTri+KL supervision scheme, our method achieves 93.53% Recall@1 and 93.9% AP on the CMHUAV dataset, with HNDM@1 (T = 50 m) exceeding 94.8% across all altitudes. Moreover, the analysis of per-altitude HNDM@1 and APW behavior confirms that the proposed model learns altitude-robust cross-view representations without requiring explicit altitude information. In terms of the evaluation metrics, we propose HNDM@K, which unifies retrieval ranking and geospatial deviation into a single evaluation framework, providing a more engineering-oriented benchmark for UAV localization tasks. Parameter sensitivity experiments further confirm that the model’s performance is stable across a range of hyperparameter choices, indicating that the reported gains are not artifacts of a specific configuration.

The proposed method still has several limitations. First, single-frame input lacks temporal constraints, leading to insufficient stability under dynamic occlusions or abrupt viewpoint changes. Second, the dataset only covers well-lit and clear weather conditions; under adverse conditions such as rain, fog, low light, motion blur, and reflective surfaces, the performance of vision-based deep models is expected to degrade due to the domain shift between training and test distributions, and this generalization capability remains to be quantified. Third, unlike traditional filtering approaches (e.g., Kalman filters) that provide analytical covariance-based uncertainty measures, the current deep-learning-based localizer lacks formal predictive uncertainty quantification.Fourth, all experiments in this study are offline image-retrieval evaluations using pre-collected UAV images and a pre-built satellite database. No closed-loop flight experiments, continuous trajectory localization, localization update-rate analysis, or accumulated drift evaluation have been conducted. Therefore, the reported accuracy indicates offline matching performance rather than system-level self-localization assurance.

Future work will therefore explore the following: (1) Temporal modeling strategies with consecutive-frame motion constraints to enforce spatio-temporal consistency. (2) A tightly coupled visual–IMU–barometer fusion framework that gracefully degrades to inertial dead-reckoning when visual confidence—measured by metrics such as the HNDM@K score—falls below a threshold. (3) Style-transfer-based generative augmentation to construct training samples under extreme weather and lighting conditions, thereby enhancing cross-condition robustness. (4) Formal uncertainty quantification for neural-network-based localizers, including Bayesian deep learning (e.g., Monte Carlo Dropout, Deep Ensembles) and evidential deep learning, to provide confidence estimates alongside each prediction. (5) While the proposed APW module effectively enhances part-level discriminability, its performance gain is contingent on the quality of the underlying chunking-based feature partitioning. In scenarios where the pre-defined annular partitions fail to align with semantically meaningful regions (e.g., highly irregular urban layouts), the learned scalar weighting may provide limited benefits. Exploring learnable, data-driven part partitions remains an important direction for future work. (6) Conduct closed-loop flight tests and real-time onboard implementation to evaluate trajectory-level localization continuity, update rate, and accumulated drift.

## Figures and Tables

**Figure 1:**
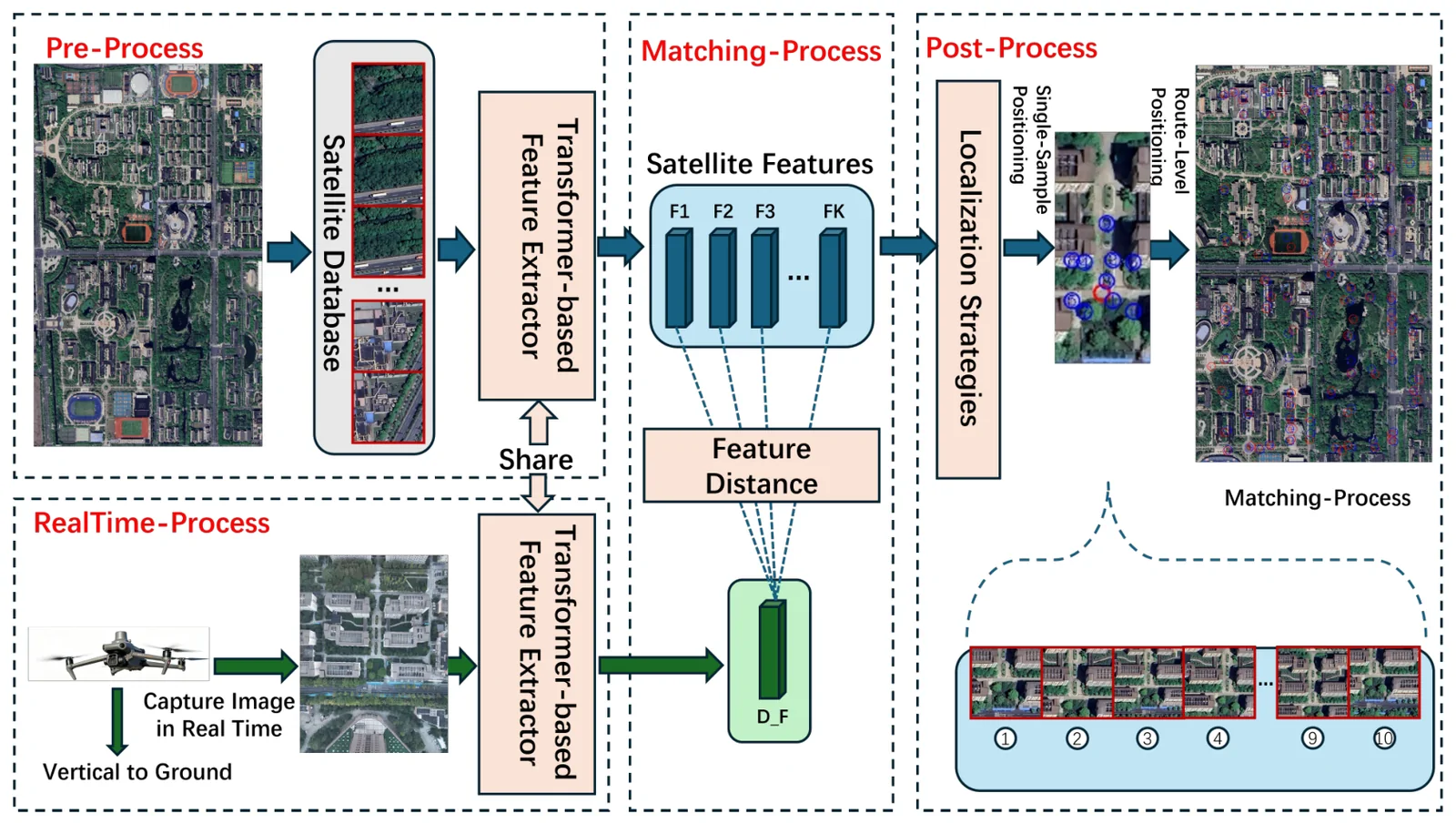
Conceptual workflow of the proposed vision-based self-localization framework. This paper evaluates the visual matching core offline; closed-loop flight validation is left for future work. There are currently two positioning strategies: global positioning and local positioning. The red and blue circles represent the true and predicted positions, respectively. In single-point prediction, the number inside the circle indicates the corresponding K value in R@K.

**Figure 2:**
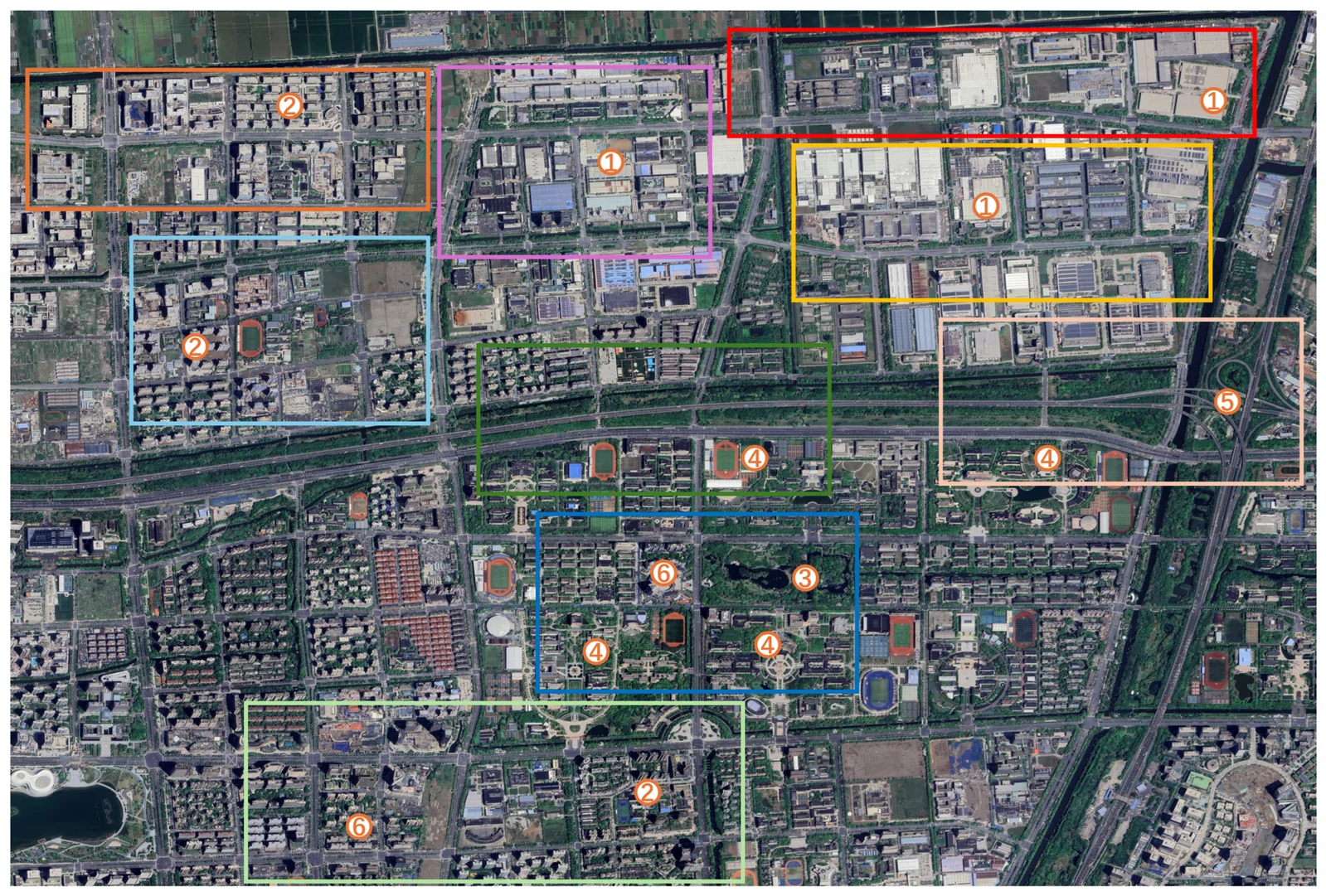
Overall locations of the image acquisition areas. Data was collected from nine adjacent, non-overlapping subregions, covering multiple scenes. Sequence 1 depicts a factory, sequence 2 a residential area, sequence 3 a park, sequence 4 a school, sequence 5 a motorway junction, and sequence 6 a commercial district.

**Figure 3:**
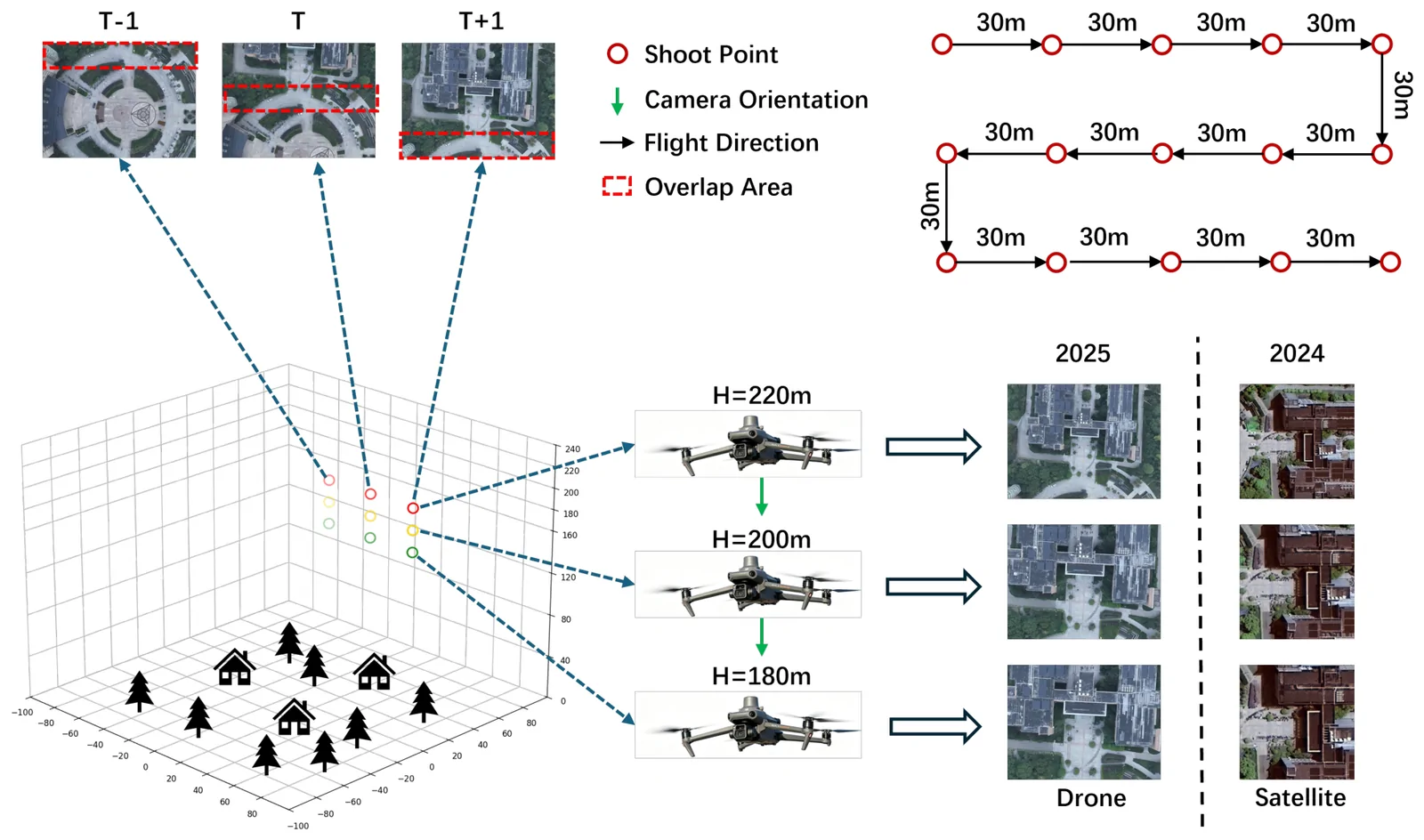
Drone flight paths, flight directions, and altitudes in the dataset.

**Figure 4:**
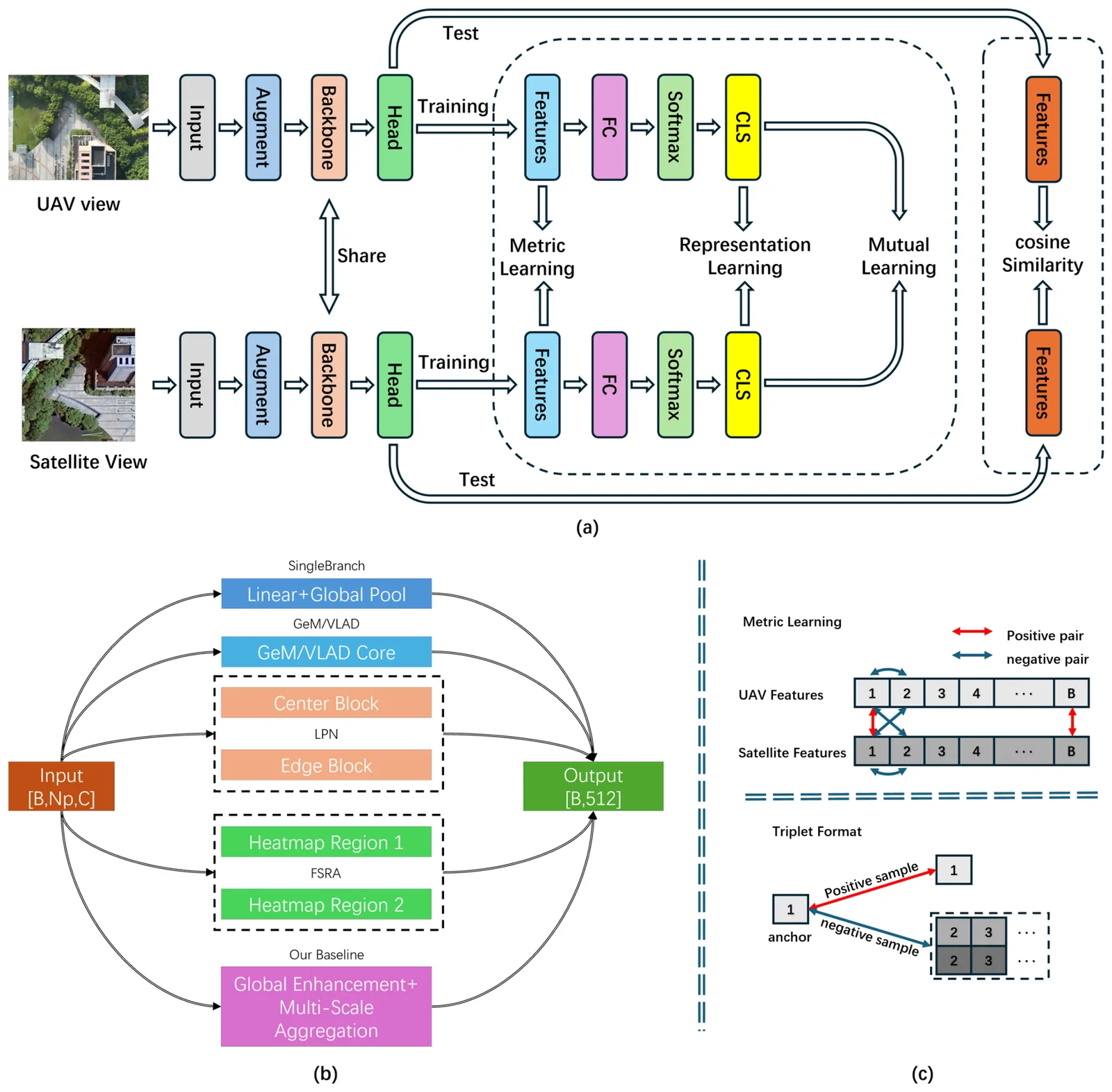
The overall architecture and core components of the model. (**a**) Training–testing workflow: the training phase employs multi-task learning for robust cross-view feature extraction, while the testing phase uses cosine similarity as the matching criterion. (**b**) A comparison of the various feature retrieval heads with the model used in this study highlights their distinct aggregation strategies; all models take ViT features as input and generate a common retrieval feature. (**c**) This figure illustrates the positive/negative sample selection and hard mining strategies employed in metric learning.

**Figure 5:**
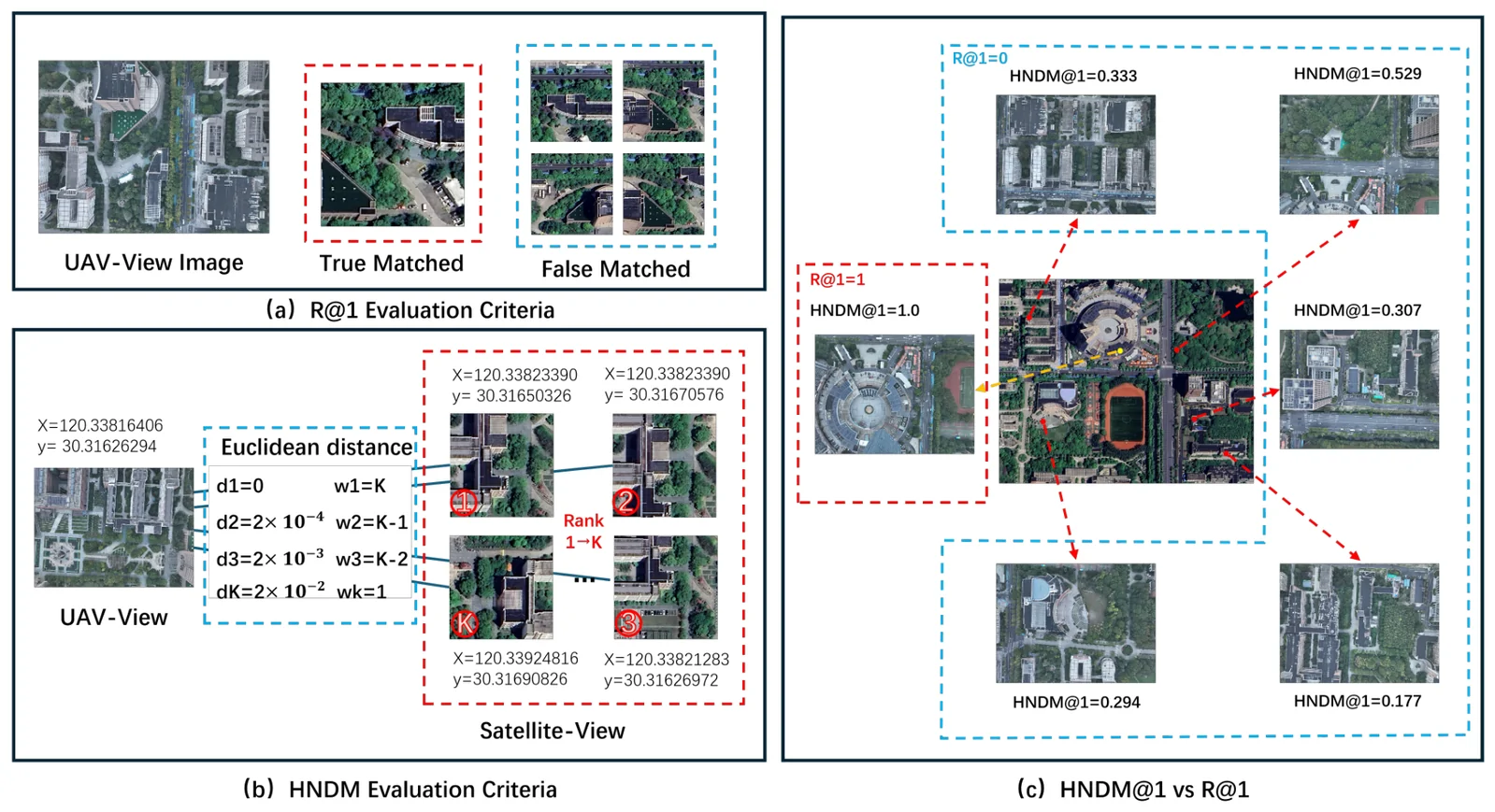
R@1 vs. HNDM@K (H = 200 m): a comparative view. (**a**) R@1 metric definition. (**b**) The distances $d_{1}$ to $d_{K}$ are the great-circle ground distances (computed via the Haversine formula) from the UAV-view query location to its *K* closest satellite image locations. The distances $d_{1}$ to $d_{K}$ and the corresponding weights $w_{1}\ldots w_{K}$ are used to compute the HNDM@K score. (**c**) Contrast of the evaluation pipelines for R@1 and HNDM@1.

**Figure 6:**
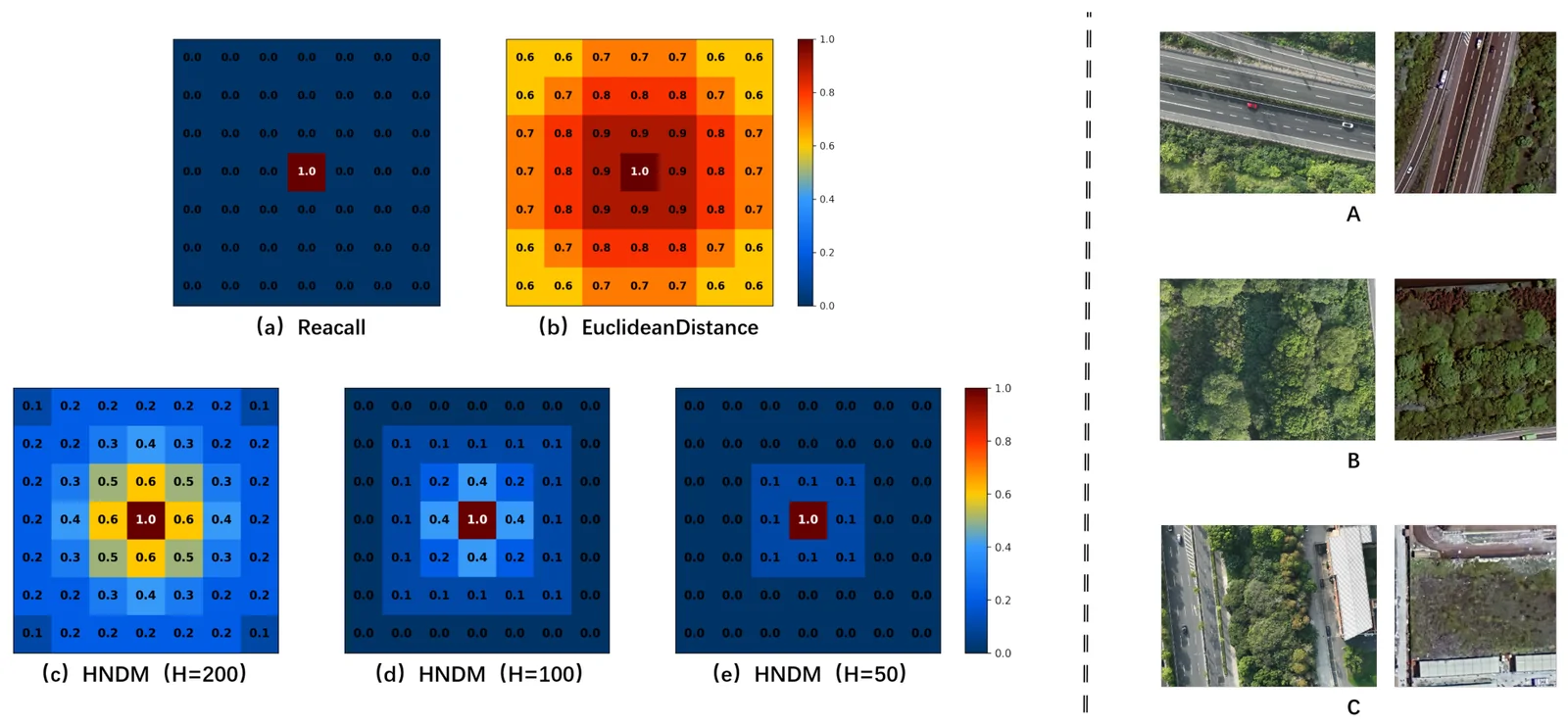
This figure illustrates (**left**) a comparative view of recall, spatial distance, and HNDM@K and (**right**) representative drone–satellite image pairs. (**A**–**C**) Three representative examples, each consisting of a UAV-view image and its corresponding satellite-view image.

**Figure 7:**
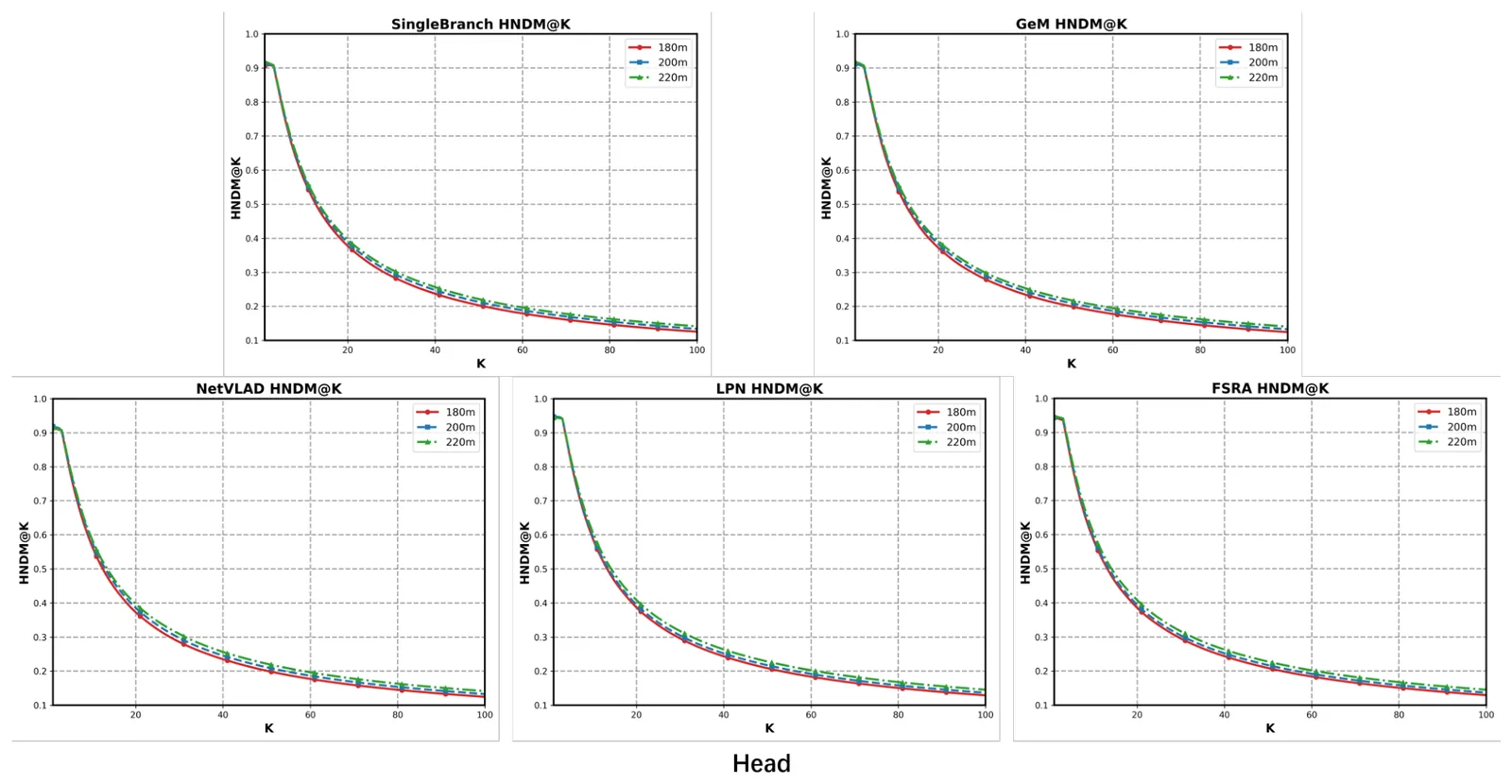
Differences between HNDM curves at three different heights with different head positions. Each subplot corresponds to one feature aggregation head; red, blue, and green curves represent 180 m, 200 m, and 220 m test heights, respectively.

**Figure 8:**
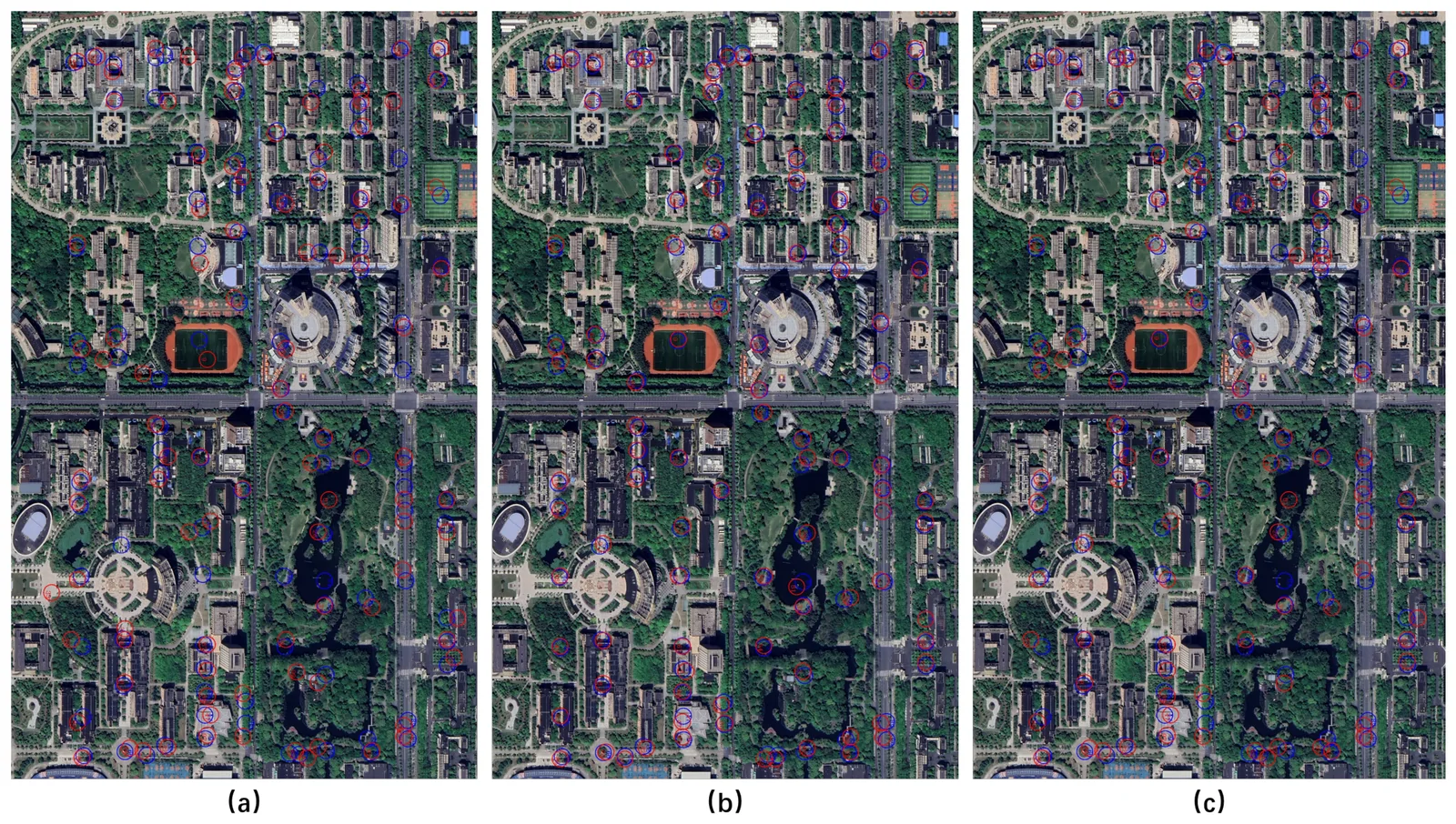
Map locations of predicted and ground-truth positions under different offline diagnostic protocols in the global positioning scenario. Red circles indicate the ground-truth position and blue circles indicate the predicted position. (**a**) R@1 predicted position and actual position. (**b**) HNDM@1 predicted position and actual position. (**c**) Predicted and actual positions for the R@1 and HNDM@1 fusion strategies.

**Figure 9:**
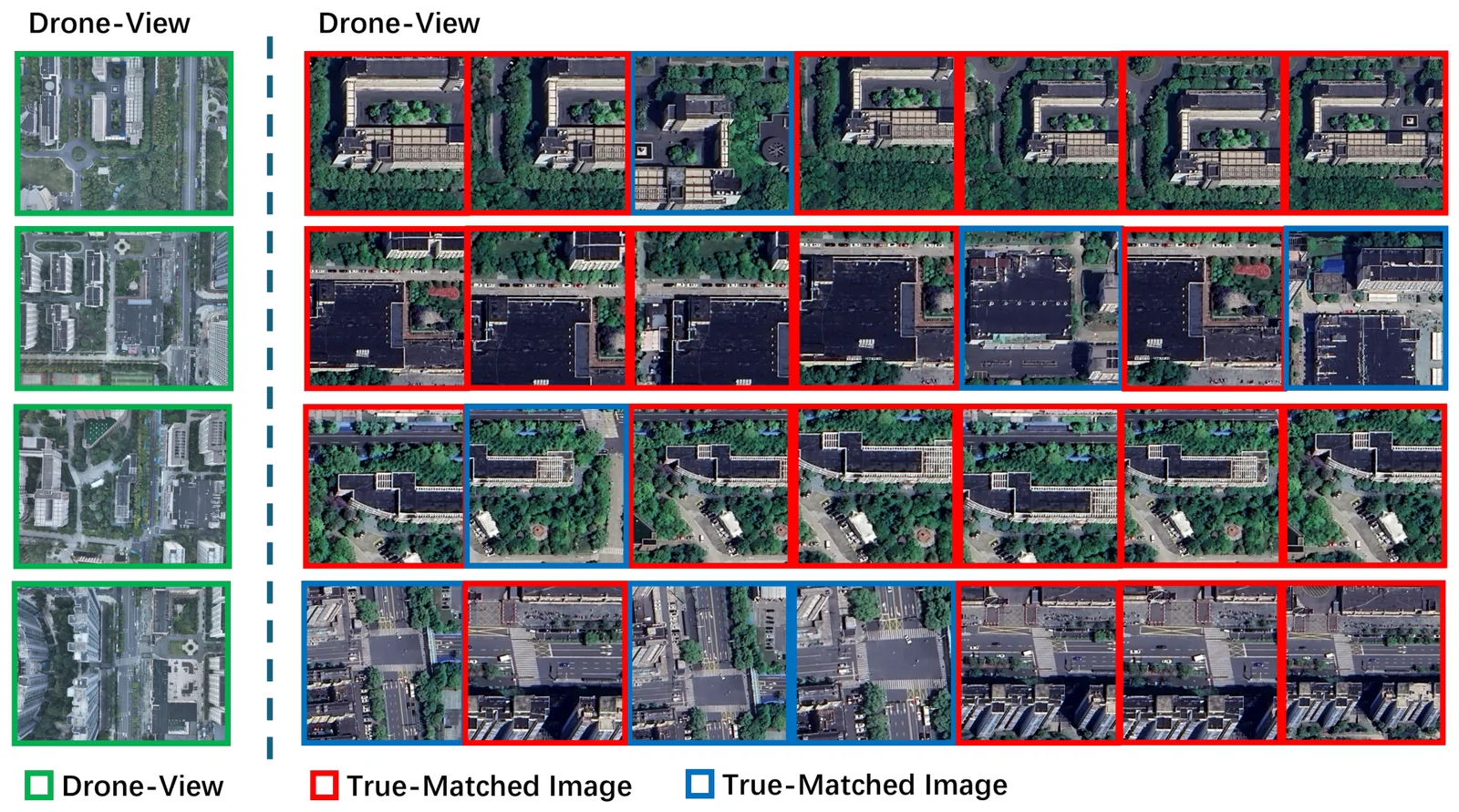
Search results for the self-localization task. The images are shown within the boxes; green indicates the query image, red indicates a correct match, and blue indicates an incorrect result.

**Figure 10:**
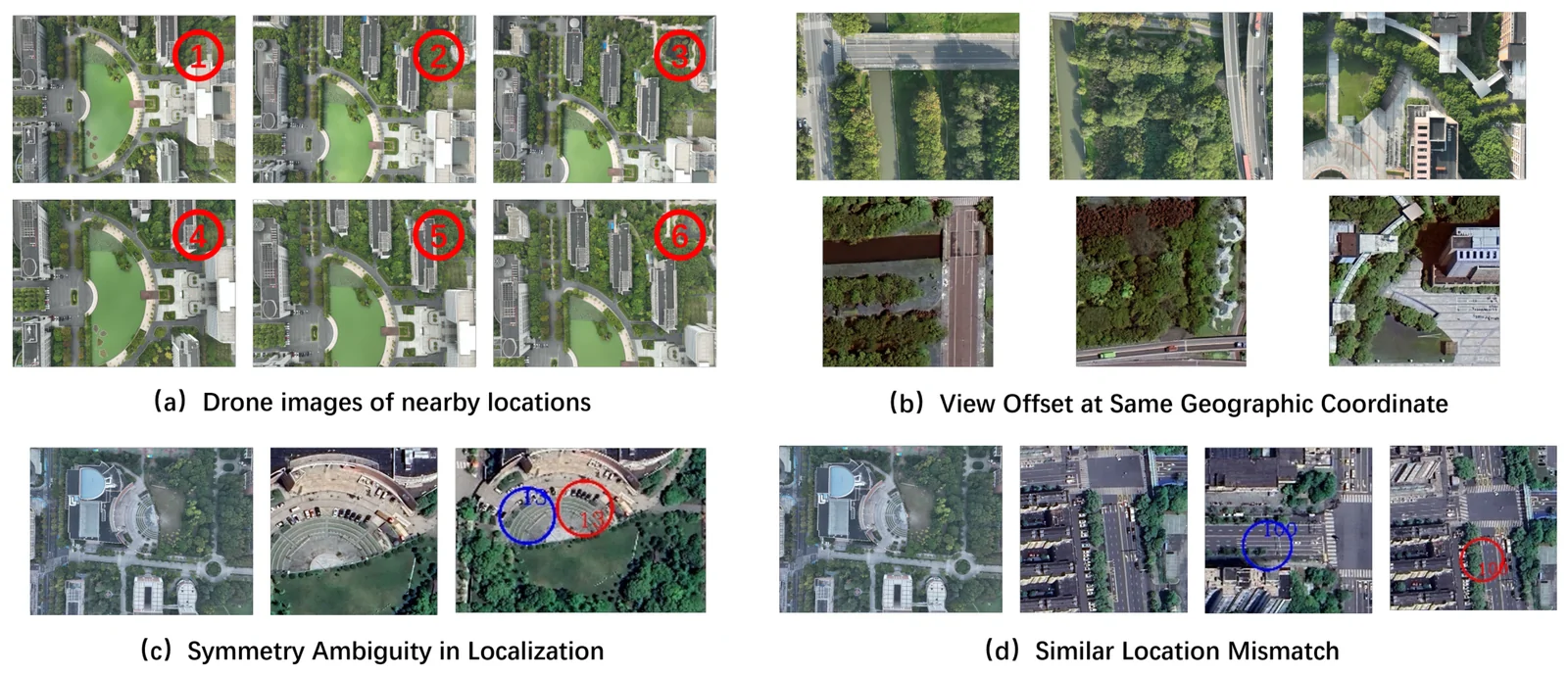
Examples of challenges in self-localization tasks. (**a**) UAV images of adjacent locations. Numbers 1–6 in (**a**) indicate six adjacent UAV-view images captured at neighboring sampling positions. (**b**) Offset in ground-level perspective while geographical coordinates remain consistent. (**c**) Symmetric ambiguity resolution. (**d**) Visual confusion due to similar positions. The red lines indicate the actual positions, while the blue lines indicate the predicted positions.

**Table 1:** Summary of existing geolocation datasets.

Datasets	Training	Platform	Images/Platform	Target	Sampling	Source	Positioning Accuracy	Evaluation
CMHUAV	6030 UAV + 6030 Sat. (2010 cls)	Drone, satellite	3 + 3	UAV	Dense	Real Scene	RTK: ≤0.1 m; combined: <1 m	R@K & AP & HNDM@K
DenseUAV	9×225.6×9	Drone, satellite	3+6	UAV	Dense	Real Scene	typically ≥1–5m	R@K & SDM@K
SUES-200	120×51	Drone, Satellite	50+1	Diverse	Discrete	Google Map	approximate, ±1–10m	R@K & AP & RB & PF
University-1652	701×71.64	Drone, Ground, Satellite	54+16.64+1	Building	Discrete	Google Map	approximate, ±1–10m	R@K & AP
VIGOR	91k+53k	Ground, Aerial	/	User	Discrete	Google Map	approximate, ±1–10m	Meter Accuracy
CVUSA	35.5k×2	Ground, Satellite	1+1	User	Discrete	Google Map	approximate, ±1–10m	R@K

**Table 2:** Dataset composition of CMHUAV.

Subset	Images		Classes	Area
	UAV-View	Satellite-View		
Training	6030	6030	2010	9
Query	2595	2595	865	9

**Table 3:** Data acquisition and annotation quality parameters of CMHUAV.

Parameter	Description
UAV platform	DJI Mavic 3T (network RTK)
RTK status filter	Fixed solution only
Horizontal positioning accuracy	≤0.1 m (RMS)
Gimbal attitude filter	Pitch deviation <$3^{\circ }$
Satellite imagery	2025 orthorectified, <0.5 m (RMSE)
Combined annotation error	<1 m (typically 0.3–0.6 m)

**Table 4:** Comparison of HNDM@1 and Recall@1 (H = 180 m, T = 50 m).

Case	Scene Description	R@1	Top-1 Distance	HNDM@1	Height
1	Correct, RTK precise alignment	1	0.0	1.0	180
2	Incorrect, spatially adjacent	0	42.5	0.79	180
3	Incorrect, completely spatially offset	0	4693	0.0	180

**Table 5:** Comparison of different methods on the CMHUAV multi-height multi-scene dataset.

Method	Backbone	R@1	AP	HNDM@1 (180 m)	HNDM@1 (200 m)	HNDM@1 (220 m)
SingleBranch (Baseline)	ViT-S	88.09%	88.47%	90.63%	91.29%	91.87%
NetVLAD	ViT-S	88.67%	88.91%	91.46%	91.85%	91.48%
GeM	ViT-S	88.67%	88.73%	91.50%	91.16%	91.70%
LPN (block = 2)	ViT-S	91.41%	92.10%	93.41%	93.78%	94.16%
FSRA (block = 2)	ViT-S	91.52%	92.12%	93.7%	94.24%	94.32%
APW (block = 2)	ViT-S	92.52%	92.97%	94.33%	94.82%	94.89%
SLUG	ViT-S	93.53%	93.90%	94.81%	95.26%	95.80%

**Table 6:** Performance comparison of models on the single-scene DenseUAV dataset.

Method	Backbone	Drone → Satellite		Satellite → Drone	
		R@1	AP	R@1	AP
SingleBranch (Baseline)	ViT-S	83.01%	72.80%	76.66%	73.96%
NetVLAD	ViT-S	84.00%	73.63%	76.60%	73.94%
GeM	ViT-S	82.58%	72.35%	77.26%	74.19%
LPN (block = 2)	ViT-S	84.51%	75.59%	78.89%	77.63%
FSRA (block = 2)	ViT-S	84.94%	75.59%	78.89%	77.31%
APW (block = 2)	ViT-S	86.57%	77.37%	81.45%	79.24%
SLUG	ViT-S	88.03%	79.57%	84.21%	81.68%

**Table 7:** Controlled re-implementation comparison with recent cross-view localization methods on CMHUAV under a unified training configuration. The reported numbers for competing methods are not their official results.

Method	Backbone	R@1	AP	HNDM@1 (180 m)	HNDM@1 (200 m)	HNDM@1 (220 m)
SAFA	ViT-S	89.06%	89.38%	91.47%	92.11%	92.54%
TransGeo	ViT-S	86.74%	87.48%	88.84%	89.56%	90.99%
GeoFormer	ViT-S	89.52%	89.89%	91.37%	92.62%	93.30%
Sample4Geo	ViT-S	76.07%	75.79%	81.27%	81.84%	82.12%
APW (block = 1)	ViT-S	92.33%	92.24%	94.44%	94.58%	94.69%

Note: All competing methods are re-implemented under the same ViT-S/224 backbone and trained with the same configuration as the SingleBranch baseline (baseline data augmentation, CE+SWTri+KL loss, and block = 1 where applicable). No method-specific hyperparameter tuning or official training recipe was used. The reported numbers are therefore not the official results of SAFA, TransGeo, GeoFormer, or Sample4Geo, and they should only be compared within this controlled re-implementation setting. SAFA is not originally a Transformer-based method but is re-implemented under the same ViT-S/224 backbone for fair comparison. APW (block = 1) denotes the proposed retrieval head under the baseline configuration; the full SLUG configuration is reported in Table 5 and in the sensitivity analysis (Section 4.4.4).

**Table 8:** Ablation study of the asymmetric data augmentation strategy. All experiments employ the same backbone (ViT-S/224), APW head, and joint loss (CE+SWTri+KL), with only the augmentation policy varied.

Augmentation Strategy	R@1	AP	HNDM@1 (180 m)	HNDM@1 (200 m)	HNDM@1 (220 m)
No Augmentation	91.02%	91.74%	93.36%	93.87%	94.02%
Symmetric Augmentation	91.25%	92.11%	93.48%	93.45%	93.83%
SLUG	93.53%	93.90%	94.81%	95.26%	95.80%

**Table 9:** Ablation study on performance gain of different loss functions.

Loss			R@1	HNDM@1 (180 m)	HNDM@1 (200 m)	HNDM@1 (220 m)
CE	SWTri	KL				
✓			89.63%	92.36%	92.46%	92.62%
	✓		81.73%	87.15%	87.45%	87.31%
		✓	2.7%	6.31%	6.93%	5.98%
	✓	✓	82.43%	87.11%	88.16%	87.35%
✓		✓	91.56%	94.03%	93.77%	94.2%
✓	✓		91.14%	93.34%	93.72%	93.78%
✓	✓	✓	93.53%	94.81%	95.26%	95.8%

**Table 10:** Parameter sensitivity analysis of SLUG on the CMHUAV dataset. All experiments use the full SLUG configuration (ViT-S/224 backbone, asymmetric augmentation, and joint loss CE+SWTri+KL), varying only the parameter under study.

Parameter	R@1	AP	HNDM@1 (180 m)	HNDM@1 (200 m)	HNDM@1 (220 m)
**(a) Number of regions (block)**					
block = 1	92.76%	92.24%	94.44%	94.58%	92.98%
block = 2 (default)	93.53%	93.90%	94.81%	95.26%	95.80%
block = 3	93.49%	93.86%	95.45%	95.37%	95.99%
**(b) Alpha in WeightedSoftTripletLoss**					
alpha = 1	91.45%	91.98%	93.17%	93.50%	94.14%
alpha = 3 (default)	93.53%	93.90%	94.81%	95.26%	95.80%
alpha = 5	92.83%	93.45%	94.76%	95.06%	94.79%

**Table 11:** Comparative evaluation of feature heads. Comparison of parameters, MACs, relative forward time, and retrieval metrics for different retrieval heads under a unified ViT-S backbone. Relative forward time is normalized to the SingleBranch baseline under identical server-side measurement conditions. It is intended only for relative comparison among retrieval heads, not as absolute latency or onboard deployment latency.

Method	Backbone	Params (M)	MACs (GFLOPs)	Relative Forward Time	R@1	AP
SingleBranch (Base)	ViT-S	22.8	8.484	1.00×	88.09%	88.47%
NetVLAD	ViT-S	22.8	8.484	0.98×	88.67%	88.91%
GeM	ViT-S	22.8	8.484	0.90×	88.67%	88.73%
LPN (block = 2)	ViT-S	25.258	8.489	1.02×	91.41%	92.10%
FSRA (block = 2)	ViT-S	25.258	8.489	0.95×	91.52%	92.12%
SLUG	ViT-S	26.488	8.491	1.10×	93.53%	93.90%

**Table 12:** Leave-one-region-out split results of SLUG on the CMHUAV dataset.

Test Region	Backbone	R@1	AP	HNDM@1 (180 m)	HNDM@1 (200 m)	HNDM@1 (220 m)
Rot1	ViT-S	80.06%	82.40%	89.53%	90.92%	91.27%
Rot2	ViT-S	80.83%	81.03%	90.28%	89.81%	87.02%
Rot3	ViT-S	88.24%	89.16%	93.46%	94.57%	94.12%
Rot4	ViT-S	88.17%	88.54%	91.96%	92.67%	92.59%
Rot5	ViT-S	78.02%	80.11%	84.20%	85.38%	85.26%
Rot6	ViT-S	74.19%	75.67%	82.36%	82.83%	83.92%
Rot7	ViT-S	72.98%	75.41%	81.47%	82.14%	82.88%
Rot8	ViT-S	87.36%	88.31%	92.52%	92.82%	93.78%
Rot9	ViT-S	78.02%	79.27%	84.39%	84.95%	85.24%
AVG	ViT-S	80.87%	82.21%	87.80%	88.45%	88.45%

**Table 13:** Leave-one-region-out split results of the baseline model on the CMHUAV dataset.

Test Region	Backbone	R@1	AP	HNDM@1 (180 m)	HNDM@1 (200 m)	HNDM@1 (220 m)
Rot1	ViT-S	78.80%	79.40%	89.18%	90.18%	86.93%
Rot2	ViT-S	80.14%	80.62%	90.93%	89.37%	88.76%
Rot3	ViT-S	86.93%	86.87%	93.87%	93.49%	93.45%
Rot4	ViT-S	87.14%	86.55%	91.91%	91.38%	91.61%
Rot5	ViT-S	77.81%	79.33%	84.74%	85.23%	86.43%
Rot6	ViT-S	68.13%	69.69%	77.05%	80.08%	80.27%
Rot7	ViT-S	69.05%	70.77%	76.67%	78.06%	79.30%
Rot8	ViT-S	88.34%	86.83%	93.29%	93.92%	94.55%
Rot9	ViT-S	77.40%	78.47%	84.83%	85.83%	84.24%
AVG	ViT-S	79.30%	79.84%	86.94%	87.50%	87.28%

**Table 14:** Offline diagnostic comparison of predicted locations within specified distance thresholds of the true locations. HNDM-guided and Fusion are oracle analyses that use ground-truth query coordinates to compute HNDM; they are not online candidate-selection strategies under GNSS-denied conditions.

Distance Threshold	1 m	3 m	5 m	10 m	20 m	30 m	50 m	100 m
R@1 (visual only)	0%	2.94%	11.76%	41.18%	75.49%	83.33%	87.25%	88.24%
HNDM-guided (offline oracle)	1.96%	11.76%	34.31%	84.31%	99.02%	100%	100%	100%
Fusion (offline oracle)	0%	3.92%	13.73%	50.00%	86.27%	93.14%	98.04%	98.04%

Note: HNDM-guided selection ranks the Top-*K* candidates by the HNDM score, whose computation requires the ground-truth UAV query coordinates to obtain $d_{i}$ in Equation (8). The Fusion strategy also relies on these coordinates. Therefore, both are offline diagnostic/oracle analyses only. HNDM@K should be used as an online criterion only when an independent query-position estimate is available.

## Data Availability

The original contributions presented in this study are included in the article. Further inquiries can be directed to the corresponding author.
